# ISSN exercise & sports nutrition review update: research & recommendations

**DOI:** 10.1186/s12970-018-0242-y

**Published:** 2018-08-01

**Authors:** Chad M. Kerksick, Colin D. Wilborn, Michael D. Roberts, Abbie Smith-Ryan, Susan M. Kleiner, Ralf Jäger, Rick Collins, Mathew Cooke, Jaci N. Davis, Elfego Galvan, Mike Greenwood, Lonnie M. Lowery, Robert Wildman, Jose Antonio, Richard B. Kreider

**Affiliations:** 1 Exercise and Performance Nutrition Laboratory, School of Health Sciences, Lindenwood University, St. Charles, MO USA; 2 Exercise & Sport Science Department, University of Mary-Hardin Baylor, Belton, TX USA; 3 School of Kinesiology, Auburn University, Auburn, AL USA; 4 Department of Exercise and Sport Science, University of North Carolina, Chapel Hill, NC USA; 5 High Performance Nutrition LLC, Mercer Island, WA USA; 6 Increnovo, LLC, Milwaukee, WI USA; 7 Collins Gann McCloskey and Barry PLLC, Mineola, NY USA; 8 Department of Health and Medical Sciences, Swinburne University of Technology, Hawthorn, Victoria Australia; 9 University of Texas Medical Branch, Galveston, TX USA; 10 Exercise & Sports Nutrition Lab, Human Clinical Research Facility, Texas A&M University, College Station, TX USA; 11 Department of Human Performance & Sport Business, University of Mount Union, Alliance, OH USA; 12 Dymatize Nutrition, LLC, Dallas, TX USA; 13 Department of Health and Human Performance, Nova Southeastern University, Davie, FL USA

**Keywords:** Sports nutrition, Performance nutrition, Position stand, Review, Recommendations, Efficacy, Double-blind, Randomized, Placebo-controlled, Dietary supplements, Ergogenic aids, Weight gain, Hypertrophy, Strength, Capacity, Power

## Abstract

**Background:**

Sports nutrition is a constantly evolving field with hundreds of research papers published annually. In the year 2017 alone, 2082 articles were published under the key words ‘sport nutrition’. Consequently, staying current with the relevant literature is often difficult.

**Methods:**

This paper is an ongoing update of the sports nutrition review article originally published as the lead paper to launch the Journal of the International Society of Sports Nutrition in 2004 and updated in 2010. It presents a well-referenced overview of the current state of the science related to optimization of training and performance enhancement through exercise training and nutrition. Notably, due to the accelerated pace and size at which the literature base in this research area grows, the topics discussed will focus on muscle hypertrophy and performance enhancement. As such, this paper provides an overview of: 1.) How ergogenic aids and dietary supplements are defined in terms of governmental regulation and oversight; 2.) How dietary supplements are legally regulated in the United States; 3.) How to evaluate the scientific merit of nutritional supplements; 4.) General nutritional strategies to optimize performance and enhance recovery; and, 5.) An overview of our current understanding of nutritional approaches to augment skeletal muscle hypertrophy and the potential ergogenic value of various dietary and supplemental approaches.

**Conclusions:**

This updated review is to provide ISSN members and individuals interested in sports nutrition with information that can be implemented in educational, research or practical settings and serve as a foundational basis for determining the efficacy and safety of many common sport nutrition products and their ingredients.

## Background

Evaluating the scientific merit of articles and advertisements about exercise and nutrition products is a key skill that all sports nutrition professionals must possess. To assist members and other advocates of the International Society of Sports Nutrition (ISSN) in keeping up to date about the latest findings in sports nutrition, the ISSN Exercise & Sports Nutrition Review: Research & Recommendations has been updated. The initial version of this paper was the first publication used to help launch the Journal of the International Society of Sports Nutrition (JISSN, originally called the Sports Nutrition Review Journal). This paper provides a definition of ergogenic aids and dietary supplements and discusses how dietary supplements are legally regulated. Other sections highlight how to evaluate the scientific merit of nutritional supplements and provide general nutritional strategies to optimize performance and enhance recovery. Finally, a brief overview of the efficacy surrounding many supplements commonly touted to promote skeletal muscle hypertrophy and improve physical performance is provided. Based upon the available scientific literature testing the efficacy and safety of the nutritional supplements discussed herein, all nutritional supplements discussed in this paper have been placed into three categories based upon the quality and quantity of scientific support available:

A) Strong Evidence to Support Efficacy and Apparently Safe
B) Limited or Mixed Evidence to Support Efficacy
C) Little to No Evidence to Support Efficacy and/or Safety

Since the last published version of this document in 2010 [1], the general approach to categorization has not changed, but several new supplements have been introduced to the market and are subsequently reviewed in this article. In this respect, many supplements have had additional studies published that has led to some supplements being placed into a different category or removed from the review altogether. We understand and expect that some individuals may not agree with our interpretations of the literature or what category we have assigned a particular supplement, but it is important to appreciate that some classifications may change over time as more research becomes available.

### Definition of an ergogenic aid

An ergogenic aid is any training technique, mechanical device, nutritional ingredient or practice, pharmacological method, or psychological technique that can improve exercise performance capacity or enhance training adaptations [2–4]. Ergogenic aids may help prepare an individual to exercise, improve exercise efficiency, enhance recovery from exercise, or assist in injury prevention during intense training. Although this definition seems rather straightforward, there is considerable debate regarding the ergogenic value of various nutritional supplements. A consensus exists to suggest that a nutritional supplement is ergogenic if peer-reviewed studies demonstrate the supplement significantly enhances exercise performance following weeks to months of ingestion (e.g., promotes increases in maximal strength, running speed, and/or work during a given exercise task). On the other hand, a supplement may also have ergogenic value if it acutely enhances the ability of an athlete to perform an exercise task or enhances recovery from a single exercise bout. The ISSN has adopted a broader view regarding the ergogenic value of supplements. While the muscle building and performance enhancing effects of a supplement on a single bout of exercise may lead to eventual ergogenic effects or optimized training adaptations, our view is that such evidence does not warrant “Excellent Evidence to Support Efficacy” if there is a lack of long-term efficacy data. Herein, we have adopted the view that a supplement is clearly ergogenic if most of human studies support the ingredient as being effective in promoting further increases in muscle hypertrophy or performance with exercise training. Conversely, supplements that fall short of this standard and are only supported by preclinical data (e.g., cell culture or rodent studies) are grouped into other categories.

### Definition and regulation of dietary supplements

#### The Dietary Supplement Health and Education Act (DSHEA) and the safety of dietary supplements

Congress passed the Dietary Supplement Health and Education Act of 1994 (DSHEA), placing dietary supplements in a special category of “foods”. In October 1994, President Clinton signed DSHEA into law. This statute was enacted amid claims that the Food and Drug Administration (FDA) was distorting the then-existing provisions of the Food, Drug, and Cosmetic Act (FDCA) to improperly deprive the public of safe and popular dietary supplement products.

The law defines a “dietary supplement” as a product that is intended to supplement the diet and contains a “dietary ingredient”. By definition, “dietary ingredients” in these products may include vitamins, minerals, herbs or other botanicals, amino acids, and substances such as enzymes, organ tissues, and glandular extracts. Further, dietary ingredients may also include extracts, metabolites, or concentrates of those substances. Dietary supplements may be found in many forms such as tablets, capsules, softgels, gelcaps, liquids, or powders, but may only be intended for oral ingestion. Dietary supplements cannot be marketed or promoted for sublingual, intranasal, transdermal, injected, or in any other route of administration except oral ingestion. A supplement can be in other forms, such as a bar, as long as the information on its label does not represent the product as a conventional food or a sole item of a meal or diet.

Indeed, DSHEA clearly defines “dietary supplements” and “dietary ingredients,” it sets certain criteria for “new dietary ingredients,” and the law prevents the FDA from overreaching. Additionally, and contrary to widespread opinion, DSHEA did not leave the industry unregulated. The dietary supplement industry is in fact regulated by the FDA as a result of DSHEA. The law ensures the authority of the FDA to provide legitimate protections for the public health. The Federal Trade Commission (FTC) also continues to have jurisdiction over the marketing claims that dietary supplement manufacturers or companies make about their products. The FDA and FTC operate in a cooperative fashion to regulate the dietary supplement industry. In this respect, the extent to which information is shared and jurisdiction between these two entities overlaps with regard to marketing and advertising dietary supplements continues to increase.

In the United States, dietary supplements are classified as food products, not drugs, and there is generally no mandate to register products with the FDA or obtain FDA approval before producing or selling supplements to consumers. However, if a dietary supplement manufacturer is making a claim about their product, the company must submit the claims to FDA within 30 days of marketing the product. Compare this, for example, with Canada where under the Natural Health Product (NHP) Regulations enacted in 2004 supplements must be reviewed, approved, and registered with Health Canada. The rationale for the U.S. model is based on a presumed long history of safe use; hence there is no need to require additional safety data.

DSHEA also requires supplement marketers to include on any label displaying structure/function claims (i.e., claims that the product affects the structure or function of the body) the mandatory FDA disclaimer *“This statement has not been evaluated by the Food and Drug Administration. This product is not intended to diagnose, treat, cure, or prevent any disease.”* Opponents of dietary supplements often cite this statement as evidence that the FDA does not review or approve dietary supplements. However, most dietary ingredients have been “grandfathered in” as DSHEA-compliant ingredients due to a long history of safe use, and those products containing new ingredients must be submitted by a notification to the FDA for a safety review prior to being brought to market. Although many dietary ingredients have been introduced into dietary supplements since October 1994 and have *not* been submitted to the FDA for a safety review, nutritional supplementation writ large is generally safe. In this regard, while there are over 50,000 dietary supplements registered with the Office of Dietary Supplement’s “Dietary Supplement Label Database”, a 2013 Annual Report (released in 2015) of the American Association of Poison Control Centers revealed zero fatalities occurred due to dietary supplements compared to 1692 deaths due to drugs [5]. Perhaps more alarming is a 2015 report by the Centers for Disease Control suggesting 2,287,273 emergency room visits were due to prescription drug-related events which dwarfs the 3266 emergency room visits due to dietary supplements (adjusted from 23,000 visits after excluding cases of older adults choking on pills, allergic reactions, unsupervised children consuming too many vitamins, and persons consuming ingredients not defined by DSHEA as a dietary supplement) [5]. Furthermore, a recent Healthcare Cost and Utilization Project Statistical Brief by Lucado et al. [6] reported approximately one in six Americans suffered from food borne illnesses in 2010, and food borne illnesses were associated with over 3.7 million treat-and-release emergency department visits, 1.3 million inpatient hospital stays, and 3000 deaths. Notwithstanding, there have been case reports of liver and kidney toxicity potentially caused by supplements containing herbal extracts [7] as well as overdoses associated with pure caffeine anhydrous ingestion [8]. Collectively, the aforementioned statistics and case reports demonstrate that while generally safe, as with food or prescription drug consumption, dietary supplement consumption can lead to adverse events in spite of DSHEA and current FDA regulations described below.

#### New dietary ingredients

Recognizing that new and untested dietary supplement products may pose unknown health issues, DSHEA distinguishes between products containing dietary ingredients that were already on the market and products containing new dietary ingredients that were not marketed prior to the enactment of the law. A “new dietary ingredient” (NDI) is defined as a dietary ingredient that was not marketed in the United States before October 15, 1994. DSHEA grants the FDA greater control over supplements containing NDIs. A product containing an NDI is deemed adulterated and subject to FDA enforcement sanctions unless it meets one of two exemption criteria: either (1) the supplement in question contains “only dietary ingredients which have been present in the food supply as an article used for food in a form in which the food has not been chemically altered”; or (2) there is a “history of use or other evidence of safety” provided by the manufacturer or distributor to the FDA at least 75 days before introducing the product into interstate commerce. The first criterion is silent as to how and by whom presence in the food supply as food articles without chemical alteration is to be established. The second criterion—applicable only to new dietary ingredients that have not been present in the food supply—requires manufacturers and distributors of the product to take certain actions. Those actions include submitting, at least 75 days before the product is introduced into interstate commerce, information that is the basis on which a product containing the new dietary ingredient is “reasonably expected to be safe.” That information would include: (1) the name of the new dietary ingredient and, if it is an herb or botanical, the Latin binomial name; (2) a description of the dietary supplement that contains the new dietary ingredient, including (a) the level of the new dietary ingredient in the product, (b) conditions of use of the product stated in the labeling, or if no conditions of use are stated, the ordinary conditions of use, and (c) a history of use or other evidence of safety establishing that the dietary ingredient, when used under the conditions recommended or suggested in the labeling of the dietary supplement, is reasonably expected to be safe.

In July 2011, the FDA released a Draft Guidance for Industry, entitled “Dietary Supplements: New Dietary Ingredient Notifications and Related Issues.” While a guidance does not carry the authority or the enforceability of a law or regulation, the FDA’s NDI draft guidance represented the agency’s current thinking on the topic. The guidance prompted great controversy, and FDA agreed to issue a revised draft guidance to address some of the issues raised by industry. In August 2016, FDA released a revised Draft Guidance that replaced the 2011 Draft Guidance. The purpose of the 2016 Draft Guidance was to help manufacturers and distributors decide whether to submit a premarket safety notification to FDA, help prepare NDI notifications in a manner that allows FDA to review and respond more efficiently and quickly, and to improve the quality of NDI notifications. The 2016 Draft Guidance has been criticized by industry and trade associations for its lack of clarity and other problems. Some of these issues include the lack of clarity regarding Pre-DSHEA, (Grandfathered), ingredients and FDA requiring an NDI notification even if another manufacturer has submitted a notification.

The lack of clarity surrounding the “new” Draft Guidance has led to many NDI notifications being rejected by FDA for lack of safety data and other issues. Other companies have opted to utilize the “Self-Affirmed Generally Recognized as Safe (GRAS)” route in order to “bypass” the NDI notification process. Self-Affirmed GRAS is when a company has a team of scientific experts evaluate the safety of their ingredient. There is no requirement that the safety dossier be submitted to FDA but is used by the company as an internal document that may be relied upon if the ingredient is challenged by the FDA. FDA has expressed its concern with this practice and does not encourage dietary supplement manufacturers to use Self-Affirmed GRAS to avoid submitting NDI notifications. In any event, the likelihood of another revised Draft Guidance from FDA becoming available in the future is high, and possibly more enforcement actions taken against companies that market an NDI without submitting a notification.

#### Adverse event reporting

In response to growing criticism of the dietary supplement industry, the 109th Congress passed the first mandatory Adverse Event Reporting (AER) legislation for the dietary supplement industry. In December 2006, President Bush signed into law the Dietary Supplement and Nonprescription Drug Consumer Protection Act, which took effect on December 22, 2007. After much debate in Congress and input from the FDA, the American Medical Association (AMA), many of the major supplement trade associations, and a host of others all agreed that the legislation was necessary and the final version was approved by all. In short, the Act requires that all “serious adverse events” regarding dietary supplements be reported to the Secretary of Health and Human Services. The law strengthens the regulatory structure for dietary supplements and builds greater consumer confidence, as consumers have a right to expect that if they report a serious adverse event to a dietary supplement marketer the FDA will be advised about it.

An adverse event is any health-related event associated with the use of a dietary supplement that is adverse. A serious adverse event is an adverse event that (A) results in (i) death, (ii) a life-threatening experience, (iii) inpatient hospitalization, (iv) a persistent or significant disability or incapacity, or (v) a congenital anomaly or birth defect; or (B) requires, based on reasonable medical judgment, a medical or surgical intervention to prevent an outcome described under subparagraph (A). Once it is determined that a serious adverse event has occurred, the manufacturer, packer, or distributor (responsible person) of a dietary supplement whose name appears on the label of the supplement shall submit to the Secretary of Health and Human Services any report received of the serious adverse event accompanied by a copy of the label on or within the retail packaging of the dietary supplement. The responsible person has 15 business days to submit the report to FDA after being notified of the serious adverse event. Following the initial report, the responsible person must submit follow-up reports of new medical information that they receive for one-year.

#### Adulterated supplements

The FDA has various options to protect consumers from unsafe supplements. The Secretary of the Department of Health and Human Services (which falls under FDA oversight) has the power to declare a dangerous supplement to be an “imminent hazard” to public health or safety and immediately suspend sales of the product. The FDA also has the authority to protect consumers from dietary supplements that do not present an imminent hazard to the public but do present certain risks of illness or injury to consumers. The law prohibits introducing adulterated products into interstate commerce. A supplement shall be deemed adulterated if it presents “a significant or unreasonable risk of illness or injury”. The standard does not require proof that consumers have actually been harmed or even that a product will harm anyone. It was under this provision that the FDA concluded that dietary supplements containing ephedra, androstenedione, and DMAA presented an unreasonable risk. Most recently, FDA imposed an importation ban on the botanical Mitragyna speciose, better known as Kratom. In 2016, FDA issued Import Alert #54–15, which allows for detention without physical examination of dietary supplements and bulk dietary ingredients that are, or contain, Kratom. Criminal penalties are present for a conviction of introducing adulterated supplement products into interstate commerce. While the harms associated with dietary supplements may pale in comparison to those linked to prescription drugs, recent pronouncements from the U.S. Department of Justice confirm that the supplement industry is being watched vigilantly to protect the health and safety of the American public.

#### Good manufacturing practices

When DSHEA was passed in 1994, it contained a provision requiring that the FDA establish and enforce current Good Manufacturing Practices (cGMPs) for dietary supplements. However, it was not until 2007 that the cGMPs were finally approved, and not until 2010 that the cGMPs applied across the industry, to large and small companies alike. The adherence to cGMPs has helped protect against contamination issues and should serve to improve consumer confidence in dietary supplements. The market improved as companies became compliant with cGMPs, as these regulations imposed more stringent requirements such as Vendor Certification, Document Control Procedures, and Identity Testing. These compliance criteria addressed the problems that had damaged the reputation of the industry with a focus on quality control, record keeping, and documentation.

However, it does appear that some within the industry continue to struggle with compliance. In Fiscal Year 2017, it was reported that approximately 23.48% of the FDA’s 656 total cGMP inspections resulted in citations for failing to establish specifications for the identity, purity, strength, and composition of dietary supplements. Further, 18.47% of those inspected were cited for failing to establish and/or follow written procedures for quality control operations. Undoubtedly, relying on certificates of analysis from the raw materials supplier without further testing, or failing to conduct identity testing of a finished product, can result in the creation of a product that contains something it should not contain such as synthetic chemicals or even pharmaceutical drugs. All members of the industry need to ensure compliance with cGMPs.

### Marketing claims

According to the 1990 Nutrition Labeling and Education Act (NLEA), the FDA can review and approve health claims (claims describing the relationship between a food substance and a reduced risk of a disease or health-related condition) for dietary ingredients and foods. However, since the law was passed it has only approved a few claims. The delay in reviewing health claims of dietary supplement ingredients resulted in a lawsuit, *Pearson v. Shalala*, filed in 1995. After years of litigation, in 1999 the U.S. Court of Appeals for the District of Columbia Circuit ruled that qualified health claims may be made about dietary supplements with approval by FDA, as long as the statements are truthful and based on adequate science. Supplement or food companies wishing to make health claims or qualified health claims about supplements can submit research evidence to the FDA for review.

The FTC also regulates the supplement industry. Unsubstantiated claims invite enforcement by the FTC (along with the FDA, state district attorney offices, groups like the Better Business Bureau, and plaintiff’s lawyers who file class action lawsuits). The FTC has typically applied a substantiation standard of “competent and reliable scientific evidence” to claims about the benefits and safety of dietary supplements. FTC case law defines “competent and reliable scientific evidence” as “tests, analyses, research, studies, or other evidence based on the expertise of professionals in the relevant area, that has been conducted and evaluated in an objective manner by persons qualified to do so, using procedures generally accepted in the profession to yield accurate and reliable results.” The FTC has claimed that this involves providing at least two clinical trials showing efficacy of the actual product, within a population of subjects relevant to the target market, supporting the structure/function claims that are made. While the exact requirements are still evolving, the FTC has acted against several supplement companies for misleading advertisements and/or structure/function claims.

### A safer industry ahead

As demonstrated, while some argue that the dietary supplement industry is “unregulated” and/or may have suggestions for additional regulation, manufacturers and distributors of dietary supplements must adhere to several federal regulations before a product can go to market. Further, before marketing products, they must have evidence that their supplements are generally safe to meet all the requirements of DSHEA and FDA regulations. For this reason, over the last 20 years, many established supplement companies have employed research and development directors who help educate the public about nutrition and exercise, provide input on product development, conduct preliminary research on products, and/or assist in coordinating research trials conducted by independent research teams (e.g., university-based researchers or clinical research sites). These companies also consult with marketing and legal teams with the responsibility to ensure structure/function claims do not misrepresent results of research findings. This has increased job opportunities for sports nutrition specialists as well as enhanced external funding opportunities for research groups interested in exercise and nutrition research.

While some companies have falsely attributed research on different dietary ingredients or dietary supplements to their own products, suppressed negative research findings, and/or exaggerated results from research studies, the trend in the sports nutrition industry has been to develop scientifically sound supplements. This trend toward greater research support is the result of: (1) attempts to honestly and accurately inform the public about results; (2) efforts to obtain data to support safety and efficacy on products for the FDA and the FTC; and/or, (3) endeavors to provide scientific evidence to support advertising claims and increase sales. While the push for more research is due in part to greater scrutiny from the FDA and FTC, it is also in response to an increasingly competitive marketplace where established safety and efficacy attracts more consumer loyalty and helps ensure a longer lifespan for the product in commerce. Companies that adhere to these ethical standards tend to prosper while those that do not will typically struggle to comply with FDA and FTC guidelines resulting in a loss of consumer confidence and an early demise for the product.

### Product development and quality assurance

A common question posed by athletes, parents, and professionals surrounding dietary supplements relates to how they are manufactured and perceived supplement quality. In several cases, established companies who develop dietary supplements have research teams who scour the medical and scientific literature looking for potentially effective nutrients. These research teams often attend scientific meetings and review the latest patents, research abstracts presented at scientific meetings, and research publications. Leading companies invest in basic research on nutrients before developing their supplement formulations and often consult with leading researchers to discuss ideas about dietary supplements and their potential for commercialization. Other companies wait until research has been presented in patents, research abstracts, or publications before developing nutritional formulations featuring the nutrient. Upon identification of new nutrients or potential formulations, the next step is to contact raw ingredient suppliers to see if the nutrient is available, if it is affordable, how much of it can be sourced and what is the available purity. Sometimes, companies develop and pursue patents involving new processing and purification processes because the nutrient has not yet been extracted in a pure form or is not available in large quantities. Reputable raw material manufacturers conduct extensive tests to examine purity of their raw ingredients. When working on a new ingredient, companies often conduct series of toxicity studies on the new nutrient once a purified source has been identified. The company would then compile a safety dossier and communicate it to the FDA as a New Dietary Ingredient submission, with the hopes of it being allowed for lawful sale.

When a powdered formulation is designed, the list of ingredients and raw materials are typically sent to a flavoring house and packaging company to identify the best way to flavor and package the supplement. In the nutrition industry, several main flavoring houses and packaging companies exist who make many dietary supplements for supplement companies. Most reputable dietary supplement manufacturers submit their production facilities to inspection from the FDA and adhere to GMP, which represent industry standards for good manufacturing of dietary supplements. Some companies also submit their products for independent testing by third-party companies to certify that their products meet label claims and that the product is free of various banned ingredients. For example, the certification service offered by NSF International includes product testing, GMP inspections, ongoing monitoring and use of the NSF Mark indicating products comply with inspection standards, and screening for contaminants. More recently, companies have subjected their products for testing by third party companies to inspect for banned or unwanted substances. These types of tests help ensure that the dietary supplement made available to athletes do not contained substances banned by the International Olympic Committee or other athletic governing bodies (e.g., NFL, NCAA, MLB, NHL, etc.). While third-party testing does not guarantee that a supplement is void of banned substances, the likelihood is reduced (e.g., Banned Substances Control Group, Informed Choice, NSF, etc.). Moreover, consumers can request copies of results of these tests and each product that has gone through testing and earned certification can be researched online to help athletes, coaches and support staff understand which products should be considered. In many situations, companies who are not willing to provide copies of test results or certificates of analysis should be viewed with caution, particularly for individuals whose eligibility to participate might be compromised if a tainted product is consumed.

### Evaluation of nutrition ergogenic aids

The ISSN recommends that potential consumers undertake a systematic process of evaluating the validity and scientific merit of claims made when assessing the ergogenic value of a dietary supplement [1, 4]. This can be accomplished by examining the theoretical rationale behind the supplement and determining whether there is any well-controlled data showing the supplement is effective. Supplements based on sound scientific rationale with direct, supportive research showing effectiveness may be worth trying or recommending. However, those based on unsound scientific results or offer little to no data supporting the ergogenic value of the actual supplement/technique may not be worthwhile. Sports nutrition specialists should be a resource to help their clients interpret the scientific and medical research that may impact their welfare and help them train more effectively. The following are recommended questions to ask when evaluating the potential ergogenic value of a supplement.

### Does the theory make sense?

Most supplements that have been marketed to improve health or exercise performance are based on theoretical applications derived from basic science or clinical research studies. Based on these preliminary studies, a dietary approach or supplement is often marketed to people proclaiming the benefits observed in these basic research studies. Although the theory may appear relevant, critical analysis of this process often reveals flaws in the scientific logic or that the claims made do not quite match up with the cited literature. By evaluating the literature one can discern whether or not a dietary approach or supplement has been based on sound scientific evidence. To do so, one is recommended to first read reviews about the training method, nutrient, or supplement from researchers who have been intimately involved in the available research and consult reliable references about nutritional and herbal supplements [1, 9]. To aid in this endeavour, the ISSN has published position statement on topics related to creatine [10], protein [11], beta-alanine [12], nutrient timing [13], caffeine [14], HMB [15], meal frequency [16], energy drinks [17], and diets and body composition [18]. Each of these documents would be excellent resources for any of these topics. In addition, other review articles and consensus statements have been published by other researchers and research groups that evaluate dietary supplements, offer recommendations on interpreting the literature, and discuss the available findings for several ingredients that are discussed in this document [19–21]. We also advise consumers to conduct a search on the nutrient, key ingredients or the supplement itself on the National Library of Medicine’s Pub Med Online (https://www.ncbi.nlm.nih.gov/pubmed/). A quick look at these references will often help determine if the theoretical impetus for supplementing with an ingredient is plausible or not. Proponents of ergogenic aids often overstate claims made about training devices and dietary supplements while opponents of ergogenic aids and dietary supplements are often either unaware or are ignorant of research supporting their use. Sports nutrition specialists have the responsibility to know the literature and search available databases to evaluate the level of merit surrounding a proposed ergogenic aid.

#### Is the supplement legal and safe?

An initial question that should be asked is whether the supplement is legal and/or safe. Some athletic associations have banned the use of various nutritional supplements (e.g., prohormones, ephedra that contains ephedrine, “muscle building” supplements, etc.) and many professional sports organization have now written language into their collective bargaining agreements that products made available by the team must be NSF certified as safe for sport. Obviously, if the supplement is banned, the sports nutrition specialist should discourage its use. In addition, many supplements lack appropriate long-term safety data. People who consider taking nutritional supplements should be well aware of the potential side effects so they can make an informed decision whether to use a supplement. Additionally, they should consult with a knowledgeable physician to see if any underlying medical problems exist that may contraindicate its use. When evaluating the safety of a supplement, it is suggested to determine if any side effects have been reported in the scientific or medical literature. In particular, we suggest determining how long a particular supplement has been studied, the dosages evaluated, and whether any side effects were observed. We also recommend consulting the Physician’s Desk Reference (PDR) for nutritional supplements and herbal supplements to see if any side effects have been reported and/or if there are any known drug interactions. If no side effects have been reported in the scientific/medical literature, we generally will view the supplement as safe for the length of time and dosages evaluated. Unfortunately, many available supplements have not had basic safety studies completed that replicate the length of time and dosages being used.

#### Is there any scientific evidence supporting the ergogenic value?

The next question to ask is whether any well-controlled data are available showing effectiveness of the proposed ergogenic aid in athletic populations or people regularly involved in exercise training. The first place to look is the list of references cited in marketing material supporting their claims. Are the abstracts or articles cited just general references or specific studies that have evaluated the efficacy of the nutrients included in the formulation or of the actual supplement? From there, one can critically evaluate the cited abstracts and articles by asking a series of questions:

- *Are the studies basic research done in animals/clinical populations or have the studies been conducted on athletes/trained subjects?* For perspective, studies reporting improved performance in rats or an individual diagnosed with type 2 diabetes may be insightful, but research conducted on non-diabetic athletes is much more practical and relevant.
- *Were the studies well controlled?* For ergogenic aid research, the gold standard study design is a randomized, double-blind, placebo controlled clinical trial. This means that neither the researcher nor the subject is aware which group received the supplement or the placebo during the study and that the subjects were randomly assigned into the placebo or supplement group. An additional element of rigor is called a cross-over design, where each subject, at different times (separated by an interval known as a “washout period”), is exposed to each of the treatments. While utilization of a cross-over design is not always feasible, it reduces the element of variability within a participant and subsequently, increases the strength of study’s findings. At times, supplement claims have been based on poorly designed studies (i.e., small groups of subjects, no control group, use of unreliable tests, etc.) or testimonials which make interpretation more difficult. Well-controlled clinical trials provide stronger evidence as to the potential ergogenic value and importantly how the findings can best be used.
- *Do the studies report statistically significant results or are claims being made on non-significant means or trends?* Appropriate statistical analysis of research results allows for an unbiased interpretation of data. Although studies reporting statistical trends may be of interest and lead researchers to conduct additional research, studies reporting statistically significant results are obviously more convincing. With this said, it is important for people to understand that oftentimes the potential effect a dietary supplement or diet regimen may have above and beyond the effect seen from the exercise bout or an accepted dietary approach is quite small. In addition, many studies examining a biochemical or molecular biology mechanism can require invasive sampling techniques or the study population being recruited is unique (very highly trained) resulting in a small number of study participants. When viewed together, the combination of these two considerations can result in statistical outcomes that do not reach statistical significance even though large mean changes were observed. In these situations, the reporting of confidence intervals on the mean change, individual responses from all participants to the investigated treatment and/or effect sizes are additional pieces of information that can allow for a more accurate interpretation. In all such cases, additional research is warranted to further examine the potential ergogenic aid before conclusions can be made.
- *Do the results of the cited studies match the claims made about the supplement or do they accurately portray the response of the supplement against an appropriate placebo or control group?* It is not unusual for marketing claims to greatly exaggerate the results found in the actual studies and do so by focusing upon just the outcomes within the supplement (treatment) group as opposed to how the supplement group changed in comparison to how a placebo group changed. Similarly, it is not uncommon for ostensibly compelling results, that may indeed be statistically significant, to be amplified while other relevant findings of significant consumer interest are obscured or omitted (e.g. a dietary supplement showing statistically significant increases in circulating testosterone yet changes in body composition or muscular performance were not superior to a placebo). The only way to determine this is to read the entire article versus focusing an entire study’s interpretation on the provided abstract or even the article citation, and compare results observed in the studies to the available marketing claims. Reputable companies accurately and completely report results of studies so that consumers can make informed decisions about using a product.
- *Were results of the study presented at a reputable scientific meeting and/or published in a peer-reviewed scientific journal?* At times, claims are based on research that has either never been published or only published in an obscure journal. The best research is typically presented at respected scientific meetings and/or published in reputable peer-reviewed journals. Three ways to determine a journal’s reputation is either: 1) identify the publisher, 2) the “impact factor” of the journal or 3) whether or not the journal is indexed and subsequently available for review on Pub Med (https://www.ncbi.nlm.nih.gov/pubmed/). Many “peer-reviewed” journals are published by companies with ties to, or are actually owned by, companies that do business with various nutritional products (even though they may be available on PubMed). Therefore, we recommend looking up the publisher’s website and see how many other journals they publish. If you see only a few other journals this is a suggestion that the journal is not a reputable journal. Additionally, one can also look up how many articles have been published by the journal in the last 6–12 months and how many of these articles are well-conducted studies. Alternatively, one can also inquire about the impact factor, a qualitative ranking determined by the number of times a journal’s articles are cited. Impact factors are determined and published by Thomson Reuters under Journal Citation Reports® (a subscription service available at most university libraries). Most journals list their impact factor on the journal home page. Historically, those articles that are read and cited the most are the most impactful scientifically.
- *Have the research findings been replicated? If so, have the results only been replicated at the same laboratory?* The best way to know an ergogenic aid works is to see that results have been replicated in several studies preferably by several separate, distinct research groups. The most reliable ergogenic aids are those in which multiple studies, conducted at different labs, have reported similar results of safety and efficacy. Additionally, replication of results by different, unaffiliated labs with completely different authors also removes or reduces the potentially confounding element of publication bias (publication of studies showing only positive results) and conflicts of interest. A notable number of studies on ergogenic aids are conducted in collaboration with one or more research scientists or co-authors that have a real or perceived economic interest in the outcome of the study. This could range from being a co-inventor on a patent application that is the subject of the ergogenic aid, being paid or receiving royalties from the creation of a dietary supplement formulation, providing consulting services for the company or having stock options or shares in a company that owns or markets the ergogenic aid described in the study. An increasing number of journals require disclosures by all authors of scientific articles, and including such disclosures in published articles. This is driven by the aim of providing greater transparency and research integrity. It is important to emphasize that disclosure of a conflict of interest does not alone discredit or dilute the merits of a research study. The primary thrust behind public disclosures of potential conflicts of interest is first and foremost transparency to the reader and second to prevent a later revelation of some form of confounding interest that has the potential of discrediting the study in question, the findings of the study, the authors, and even the research center or institution where the study was conducted.

### Classifying and categorizing supplements

Dietary supplements may contain carbohydrate, protein, fat, minerals, vitamins, herbs, enzymes, metabolic intermediates (i.e., select amino acids), or various plant/food extracts. Supplements can generally be classified as convenience supplements (e.g., energy bars, gels, blocks, meal replacement powders, or ready to drink supplements) designed to provide a convenient means of meeting necessary energy or macronutrient needs while also providing support towards attempts at managing caloric intake, weight gain, weight loss, and/or performance enhancement. As discussed previously, evaluating the available scientific literature is an important step in determining the efficacy of any diet, diet program or dietary supplement. In considering this, nutritional supplements can be categorized in the following manner:

I. **Strong Evidence to Support Efficacy and Apparently Safe:** Supplements that have a sound theoretical rationale with the majority of available research in relevant populations using appropriate dosing regimens demonstrating both its efficacy and safety.
II. **Limited or Mixed Evidence to Support Efficacy:** Supplements within this category are characterized as having a sound scientific rationale for its use, but the available research has failed to produce consistent outcomes supporting its efficacy. Routinely, these supplements require more research to be completed before researchers can begin to understand their impact. Importantly, these supplements have no available evidence to suggest they lack safety or should be viewed as harmful.
III. **Little to No Evidence to Support Efficacy and/or Safety:** Supplements within this category generally lack a sound scientific rationale and the available research consistently shows it to lack efficacy. Alternatively, supplements that may be harmful to one’s health or to lack safety are also placed in this category.

Several factors are evaluated when beginning to counsel individuals who regularly complete exercise training. First, a clear understanding of the athlete’s goals and the time with which they have to meet those goals is important. In addition to monitoring load and recovery, an evaluation of the individual’s diet and training program should also be completed. To accomplish this, one should make sure the athlete is eating an energy balanced, nutrient dense diet that meets their estimated daily energy needs and that they are training intelligently. Far too many athletes or coaches focus too heavily upon supplementation or applications of supplementation and neglect these key fundamental aspects. Following this, we suggest that they generally only recommend supplements in category I (i.e., ‘Strong Evidence to Support Efficacy and Apparently Safe’). If an athlete is interested in trying supplements in category II (i.e., ‘Limited or Mixed Evidence to Support Efficacy’), the athlete should make sure they understand these supplements are more experimental and they may or may not see the type of results claimed. Obviously, the ISSN does not support athletes taking supplements in category III (i.e., ‘Little to No Evidence to Support Efficacy and/or Safety’). We believe this approach is scientifically substantiated and offers a balanced view as opposed to simply dismissing the use of all dietary supplements.

### General dietary guidelines for active individuals

A well-designed diet that meets energy intake needs and incorporates proper timing of nutrients is the foundation upon which a good training program can be developed [22, 23]. Research has clearly shown that lacking sufficient calories and/or enough of the right type of macronutrients may impede an athlete’s training adaptations, while athletes who consume a balanced diet that meets energy needs can augment physiological training adaptations. Moreover, maintaining an energy deficient diet during training may lead to loss of muscle mass, strength, and bone mineral density in addition to an increased susceptibility to illness and injuries, disturbances in immune, endocrine and reproductive function, and an increased prevalence of overreaching and/or overtraining. Incorporating good dietary practices as part of a training program is one way to help optimize training adaptations and prevent overtraining. The following is an overview of energy intake recommendations and major nutrient needs for active individuals.

#### Energy needs

The primary component to optimize training and performance through nutrition is to ensure the athlete is consuming enough calories to offset energy expenditure [22–26]. People who participate in a general fitness program (e.g., exercising 30–40 min per day, 3 times per week) can typically meet nutritional needs following a normal diet (e.g., 1800–2400 kcals/day or about 25–35 kcals/kg/day for a 50–80 kg individual) because their caloric demands from exercise are not too great (e.g., 200–400 kcals/session). However, athletes involved in moderate levels of intense training (e.g., 2–3 h per day of intense exercise performed 5–6 times per week) or high volume intense training (e.g., 3–6 h per day of intense training in 1–2 workouts for 5–6 days per week) may expend 600–1200 kcals or more per hour during exercise [24]. For this reason, their caloric needs may approach 40–70 kcals/kg/day (2000–7000 kcals/day for a 50–100 kg athlete). For elite athletes, energy expenditure during heavy training or competition will further exceed these levels [27, 28]. For example, energy expenditure for cyclists to compete in the Tour de France has been estimated as high as 12,000 kcals/day (150–200 kcals/kg/day for a 60–80 kg athlete) [29, 30]. Additionally, caloric needs for large athletes (i.e., 100–150 kg) may range between 6000 and 12,000 kcals/day depending on the volume and intensity of different training phases [31].

Although some argue that athletes can meet caloric needs simply by consuming a well-balanced diet, it is often very difficult for larger athletes and athletes engaged in high volume/intense training to be able to eat enough food, on a daily basis, to meet caloric needs [2, 29, 30, 32–34]. This point was clearly highlighted in a review by Burke who demonstrated that carbohydrate needs are largely unmet by high-level athletes [22]. Additionally it is difficult to consume enough food and maintain gastrointestinal comfort to train or race at peak levels [35]. Maintaining an energy deficient diet during training often leads to a number of physical (i.e., loss of fat-free mass, illness, reduced sleep quality, incomplete recovery, hormonal fluctuations, increased resting heart rate, etc.) and psychological (i.e., apathy towards training, heightened stress) adverse outcomes [23, 27]. Nutritional analyses of athletes’ diets have revealed that many are susceptible to maintaining negative energy intakes during training. It is still a question whether there may be specific individualized occasions when negative energy balance may enhance performance in the days prior to running performance [36]. Populations susceptible to negative energy balance include runners, cyclists, swimmers, triathletes, gymnasts, skaters, dancers, wrestlers, boxers, and athletes attempting to lose weight too quickly [37]. Additionally, female athletes are at particular risk of under fueling due to both competitive and aesthetic demands of their sport and their surrounding culture. Female athletes have been reported to have a high incidence of eating disorders [38]. Low or reduced energy availability (LEA) is linked to functional hypothalamic oligomenorrhea/amenorrhea (FHA), which is frequently reported in weight sensitive sports. This makes LEA a major nutritional concern for female athletes [39]. Consequently, it is important for the sports nutrition specialist working with athletes to assess athletes individually to ensure that athletes are well fed according to the goals of their sport and their health, and consume enough calories to offset the increased energy demands of training, and maintain body weight. Although this sounds relatively simple, intense training often suppresses appetite and/or alters hunger patterns so that many athletes do not feel like eating [37, 38]. Some athletes prefer not to exercise within several hours after eating because of sensations of fullness and/or a predisposition to cause gastrointestinal distress. Further, travel and training schedules may limit food availability or the types of food athletes are accustomed to eating. This means that care should be taken to plan meal times in concert with training, as well as to make sure athletes have sufficient availability of nutrient dense foods throughout the day for snacking between meals (e.g., fluids, carbohydrate/protein-rich foods and supplemental bars, etc.) [2, 33, 40]. For this reason, sports nutritionists’ often recommend that athletes consume four to six meals per day and snacks in between meals to meet energy needs. Due to these practical concerns, the use of nutrient dense energy foods, energy bars and high calorie carbohydrate/protein supplements provides a convenient way for athletes to supplement their diet in order to maintain energy intake during training.

#### Carbohydrate

Beyond optimal energy intake, consuming adequate amounts of carbohydrate, protein, and fat is important for athletes to optimize their training and performance. In particular and as it relates to exercise performance, the need for optimal carbohydrates before, during and after intense and high-volume bouts of training and competition is evident [41]. Excellent reviews [42, 43] and original investigations [44–49] continue to highlight the known dependence on carbohydrates that exists for athletes competing to win various endurance and team sport activities. A complete discussion of the needs of carbohydrates and strategies to deliver optimal carbohydrate and replenish lost muscle and liver glycogen extend beyond the scope of this paper, but the reader is referred to several informative reviews on the topic [23, 41, 50–53].

As such, individuals engaged in a general fitness program and are not necessarily training to meet any type of performance goal can typically meet daily carbohydrate needs by consuming a normal diet (i.e., 45–55% CHO [3–5 g/kg/day], 15–20% PRO [0.8–1.2 g/kg/day], and 25–35% fat [0.5–1.5 g/kg/day]). However, athletes involved in moderate and high-volume training need greater amounts of carbohydrate and protein (discussed later) in their diet to meet macronutrient needs [50]. In terms of carbohydrate needs, athletes involved in moderate amounts of intense training (e.g., 2–3 h per day of intense exercise performed 5–6 times per week) typically need to consume a diet consisting of 5–8 g/kg/day or 250–1200 g/day for 50–150 kg athletes of carbohydrate to maintain liver and muscle glycogen stores [23, 24, 50]. Research has also shown that athletes involved in high volume intense training (e.g., 3–6 h per day of intense training in 1–2 daily workouts for 5–6 days per week) may need to consume 8–10 g/day of carbohydrate (i.e., 400–1500 g/day for 50–150 kg athletes) in order to maintain muscle glycogen levels [50]. Preferably, the majority of dietary carbohydrate should come from whole grains, vegetables, fruits, etc. while foods that empty quickly from the stomach such as refined sugars, starches and engineered sports nutrition products should be reserved for situations in which glycogen resynthesis needs to occur at accelerated rates [53]. In these situations, the absolute delivery of carbohydrate (> 8 g of carbohydrate/kg/day or at least 1.2 g of carbohydrate/kg/hour for the first four hours into recovery) takes precedence over other strategies such as those that may relate to timing or concomitant ingestion of other macronutrients (e.g., protein) or non-nutrients (e.g., caffeine) or carbohydrate type (i.e., glycemic index) [50].

When considering the carbohydrate needs throughout an exercise session, several key factors should be considered. Previous research has indicated athletes undergoing prolonged bouts (2–3 h) of exercise training can oxidize carbohydrates at a rate of 1–1.1 g per minute or about 60 g per hour [41]. Several reviews advocate the ingestion of 0.7 g of carbohydrate/kg/hr. during exercise in a 6–8% solution (i.e., 6–8 g per 100 ml of fluid) [41, 42, 50, 54]. It is now well established that different types of carbohydrates can be oxidized at different rates in skeletal muscle due to the involvement of different transporter proteins that result in carbohydrate uptake [55–59]. Interestingly, combinations of glucose and sucrose or maltodextrin and fructose have been reported to promote greater exogenous rates of carbohydrate oxidation when compared to situations when single sources of carbohydrate are ingested [55–63]. These studies generally indicate a ratio of 1–1.2 for maltodextrin to 0.8–1.0 fructose seems to support the greatest rates of carbohydrate oxidation during exercise. Additional research on high molecular weight amylopectin indicates that there may be a benefit to the lower osmolality of the starch, allowing for greater consumption (100 g/hour) and possibly greater oxidation rates and performance improvement [64–67]. In addition to oxidation rates and carbohydrate types, the fasting status and duration of the exercise bout also function as key variables for athletes and coaches to consider. When considering duration, associated reviews have documented that bouts of moderate to intense exercise need to reach exercise durations that extend well into 90th minute of exercise before carbohydrate is shown to consistently yield an ergogenic outcome [41, 68, 69]. Of interest, however, not all studies indicate that shorter (60–75 min) bouts of higher intensity work may benefit from carbohydrate delivery. Currently the mechanisms surrounding these findings are, respectively, thought to be replacement of depleted carbohydrate stores during longer duration of moderate intensity while benefits seen during shorter, more intense exercise bouts are thought to operate in a central fashion. Moreover, these reviews have also pointed to the impact of fasting status on documentation of ergogenic outcomes [41, 68, 69]. In this respect, when studies require study participants to commence exercise in a fasted state, ergogenic outcomes are more consistently reported, yet other authors have questioned the ecological validity of this approach for competing athletes [43].

As it stands, the need for optimal carbohydrates in the diet for those athletes seeking maximal physical performance is unquestioned. Daily consumption of appropriate amounts of carbohydrate is the first and most important step for any competing athlete. As durations extend into 2 h, the need to deliver carbohydrate goes up, particularly when commencing exercise in a state of fasting or incomplete recovery. Once exercise ceases, several dietary strategies can be considered to maximally replace lost muscle and liver glycogen, particularly if a limited window of recovery exists. In these situations, the first priority should lie with achieving aggressive intakes of carbohydrate while strategies such as ingesting protein with lower carbohydrate amounts, carbohydrate and caffeine co-ingestion or certain forms of carbohydrate may also help to facilitate rapid assimilation of lost glycogen.

#### Protein

Considerable debate exists surrounding the amount of protein needed in an athlete’s diet [70–74]. Initially, it was recommended that athletes do not need to ingest more than the RDA for protein (i.e., 0.8 to 1.0 g/kg/d for children, adolescents and adults). However, research spanning the past 30 years has indicated that athletes engaged in intense training may benefit from ingesting about two times the RDA of protein in their diet (1.4–1.8 g/kg/d) to maintain protein balance [11, 70, 71, 73, 75–80]. If an insufficient amount of protein is consumed, an athlete will develop and maintain a negative nitrogen balance, indicating protein catabolism and slow recovery. Over time, this may lead to muscle wasting, injuries, illness, and training intolerance [76, 77, 81].

For people involved in a general fitness program or simply interested in optimizing their health, recent research suggests protein needs may also be above the RDA. Phillips and colleagues [76], Witard et al. [82], Jager et al. [11] and Tipton et al. [79] report that current evidence indicates optimal protein intakes in the range of 1.2–2.0 g/kg/day should be considered. In this respect, Morton and investigators [83] performed a meta-review and meta-regression involving 49 studies and 1863 participants and concluded that a daily protein intake of 1.62 g/kg/day may be an ideal place to start, with intakes beyond that providing no further contribution to increases in fat-free mass. In addition and in comparison to the RDA, non-exercising, older individuals (53–71 years) may also benefit from a higher daily protein intake (e.g., 1.0–1.2 g/kg/day of protein). Recent reports suggest that older muscle may be slower to respond and less sensitive to protein ingestion, typically requiring 40 g doses to robustly stimulate muscle protein synthesis [84–86]. Studies in younger individuals, however, have indicated that in the absence of exercise, a 20 g dose can maximize muscle protein synthesis [87, 88] and if consumed after a multiple set workout consisting of several exercises that target large muscle groups a 40 g dose might be needed [89]. Consequently, it is recommended that athletes involved in moderate amounts of intense training consume 1.2–2.0 g/kg/day of protein (60–300 g/day for a 50–150 kg athlete) while athletes involved in high volume, intense training consume 1.7–2.2 g/kg/day of protein (85–330 g/day for a 50–150 kg athlete) [78, 90]. This protein need would be equivalent to ingesting 3–15 three-ounce servings of chicken or fish per day for a 50–150 kg athlete [78]. Although smaller athletes typically can ingest this amount of protein, on a daily basis, in their normal diet, larger athletes often have difficulty consuming this much dietary protein. Additionally, a number of athletic populations are known to be susceptible to protein malnutrition (e.g., runners, cyclists, swimmers, triathletes, gymnasts, dancers, skaters, wrestlers, boxers, etc.) and consequently, additional counseling and education may be needed to help these athletes meet their daily protein needs. To this point, the periods of energy restriction to meet weight or aesthetic demands of their sports that are seemingly a part of the sport’s fabric creates an arguably greater need to understand that protein intake, quality and timing as well as combination with carbohydrate is particularly important to maintain lean body mass, training effects, and performance [25]. Overall, it goes without saying that care should be taken to ensure that athletes consume a sufficient amount of quality protein in their diet to maintain nitrogen balance.

Proteins differ based on their source, amino acid profile, and the methods of processing or isolating the protein undergoes [11]. These differences influence the availability of amino acids and peptides, which may possess biological activity (e.g., α-lactalbumin, ß-lactoglobulin, glycomacropeptides, immunoglobulins, lactoperoxidases, lactoferrin, etc.). Additionally, the rate of digestion and/or absorption and metabolic activity of the protein also are important considerations [91]. For example, different types of proteins (e.g., casein, whey, and soy) are digested at different rates, which may affect whole body catabolism and anabolism and acute stimulation of muscle protein synthesis (MPS) [91–96]. Therefore, care should be taken not only to make sure the athlete consumes enough protein in their diet but also that the protein is high quality. The best dietary sources of low fat, high quality protein are light skinless chicken, fish, egg whites, very lean cuts of beef and skim milk (casein and whey) while protein supplements routinely contain whey, casein, milk and egg protein. In what is still an emerging area of research, various plant sources of protein have been examined for their ability to stimulate increases in muscle protein synthesis [77, 97] and promote exercise training adaptations [98]. While amino acid absorption from plant proteins is generally slower, leucine from rice protein has been found to be absorbed even faster than from whey [99], while digestive enzymes [100], probiotics [101] and HMB [102] can be used to overcome differences in protein quality. Preliminary findings suggest that rice [98] and pea protein [103] may be able to stimulate similar changes in fat-free mass and strength as whey protein, although the reader should understand that many other factors (dose provided, training status of participants, duration of training and supplementation, etc.) will ultimately impact these outcomes and consequently more research is needed.

While many reasons and scenarios exist for why an athlete may choose to supplement their diet with protein powders or other forms of protein supplements, this practice is not considered to be an absolute requirement for increased performance and adaptations. Due to nutritional, societal, emotional and psychological reasons, it is preferable for the majority of daily protein consumed by athletes to occur as part of a food or meal. However, we recognize and embrace the reality that situations commonly arise where efficiently delivering a high-quality source of protein takes precedence. Jager and colleagues [11] published an updated position statement of the International Society of Sports Nutrition that is summarized by the following points:

1. An acute exercise stimulus, particularly resistance exercise and protein ingestion both stimulate muscle protein synthesis (MPS) and are synergistic when protein consumption occurs before or after resistance exercise
2. For building and maintaining muscle mass, an overall daily protein intake of 1.4–2.0 g/kg/d is sufficient for most exercising individuals
3. Higher protein intakes (2.3–3.1 g/kg fat-free mass/d) may be needed to maximize the retention of lean body weight in resistance trained subjects during hypocaloric periods
4. Higher protein intakes (> 3.0 g protein/kg body weight/day) when combined with resistance exercise may have positive effects on body composition in resistance trained individuals (i.e., promote loss of fat mass)
5. Optimal doses for athletes to maximize MPS are mixed and are dependent upon age and recent resistance exercise stimuli. General recommendations are 0.25–0.55 g of a high-quality protein per kg of body weight, or an absolute dose of 20–40 g.
6. Acute protein doses should contain 700–3000 mg of leucine and/or a higher relative leucine content, in addition to a balanced array of the essential amino acids (EAAs)
7. Protein doses should ideally be evenly distributed, every 3–4 h, across the day
8. The optimal time period during which to ingest protein is likely a matter of individual tolerance; however, the anabolic effect of exercise is long-lasting (at least 24 h), but likely diminishes with increasing time post-exercise
9. Rapidly digested proteins that contain high proportions of EAAs and adequate leucine, are most effective in stimulating MPS
10. Different types and quality of protein can affect amino acid bioavailability following protein supplementation; complete protein sources deliver all required EAAs

#### Fat

The dietary recommendations of fat intake for athletes are similar to or slightly greater than dietary recommendations made to non-athletes to promote health. Maintenance of energy balance, replenishment of intramuscular triacylglycerol stores and adequate consumption of essential fatty acids are important for athletes, and all serve as reasons for an increased intake of dietary fat [104]. Depending upon the athlete’s training status or goals, the amount of dietary fat recommended for daily intake can change. For example, higher-fat diets appear to maintain circulating testosterone concentrations better than low-fat diets [105–107]. Additionally, higher fat intakes may provide valuable translational evidence to the documented testosterone suppression which can occur during volume-type overtraining [108]. Generally, it is recommended that athletes consume a moderate amount of fat (approximately 30% of their daily caloric intake), while proportions up to 50% of daily calories can be safely ingested by athletes during regular high-volume training [104]. In situations where an athlete may be interested in reducing their body fat, dietary fat intakes ranging from 0.5 to 1 g/kg/day have been recommended results in situations where daily fat intake might comprise as little as 20% of total calories in the diet [2]. This recommendation stems largely from available evidence in weight loss studies involving non-athletic individuals that people who are most successful in losing weight and maintaining the weight loss are those who ingest reduced amounts of fat in their diet [109, 110] although this is not always the case [111]. Strategies to help athletes manage dietary fat intake include teaching them which foods contain various types of fat so that they can make better food choices and how to count fat grams [2, 33].

For years, high-fat diets have been used by athletes with the majority of evidence showing no ergogenic benefit and consistent gastrointestinal challenges [112]. In recent years, significant debate has swirled regarding the impact of increasing dietary fat. One strategy, “train low, compete high”, refers to an acute pattern of dietary periodization whereby an athlete first follows a high-fat, low carbohydrate diet for one to 3 weeks while training before reintroducing carbohydrates back into the diet. While intramuscular adaptations result that may theoretically impact performance [113, 114], no consistent, favorable impact on performance has been documented [112, 115]. A variant of high-fat diets, ketogenic diets, have increased in popularity. While no exact prescription exists, nearly all ketogenic diet prescriptions derive at least 70–80% of their daily calories from dietary fat, prescribe a moderate amount of protein (20–25% total calories or 2.0–2.5 g/kg/day) and are largely devoid of carbohydrate (10–40 g per day). This diet prescription leads to a greater reliance on ketones as a fuel source. Currently, limited and mixed evidence remains regarding the overall efficacy of a ketogenic diet for athletes. In favor, Cox et al. [116] demonstrated that ketogenic dieting can improve exercise endurance by shifting fuel oxidation while Burke and colleagues [115] failed to show an increase in performance in a cohort of Olympic-caliber race walkers. Additionally, Jabekk and colleagues [117] reported decreases in body fat with no change in lean mass in overweight women who resistance trained for 10 weeks and followed a ketogenic diet. In light of the available evidence being limited and mixed, more human research needs to be completed before appropriate recommendations can be made towards the use of high fat diets for athletic performance.

#### Strategic eating and refueling

In addition to the general nutritional guidelines described above, research has also demonstrated that timing and composition of meals consumed may play a role in optimizing performance, training adaptations, and preventing overtraining [2, 25, 40]. In this regard, it takes about 4 h for carbohydrate to be digested and assimilated into muscle and liver tissues as glycogen. Consequently, pre-exercise meals should be consumed about four to 6 h before exercise [40]. This means that if an athlete trains in the afternoon, breakfast can be viewed to have great importance to top off muscle and liver glycogen levels. Research has also indicated that ingesting a light carbohydrate and protein snack 30 to 60 min prior to exercise (e.g., 50 g of carbohydrate and 5 to 10 g of protein) serves to increase carbohydrate availability toward the end of an intense exercise bout [118, 119]. This also serves to increase availability of amino acids, decrease exercise-induced catabolism of protein, and minimize muscle damage [120–122]. Additionally, athletes who are going through periods of energy restriction to meet weight or aesthetic demands of sports should understand that protein intake, quality and timing as well as combination with carbohydrate is particularly important to maintain lean body mass, training effects, and performance [25]. When exercise lasts more than 1 h and especially as duration extends beyond 90 min, athletes should ingest glucose/electrolyte solutions (GES) to maintain blood glucose levels, prevent dehydration, and reduce the immunosuppressive effects of intense exercise [40, 123–128]. Notably, this strategy becomes even more important if the athlete is under-fueled prior to the exercise task or is fasted vs. unfasted at the start of exercise [68, 69, 129]. Following intense exercise, athletes should consume carbohydrate and protein (e.g., 1 g/kg of carbohydrate and 0.5 g/kg of protein) within 30 min after exercise and consume a high carbohydrate meal within 2 h following exercise [2, 74]. This nutritional strategy has been found to accelerate glycogen resynthesis as well as promote a more anabolic hormonal profile that may hasten recovery [120, 130, 131], but as mentioned above only when rapid glycogen restoration is needed or if the carbohydrate intake in the diet is adequate (< 6 g/kg/day) [53, 132]. In other words, the total carbohydrate consumption and timing of carbohydrate consumption should be individualized to each athlete’s needs according to the goals of the training cycle and bout [112]. Finally, for two to 3 days prior to competition, athletes should taper training by 30 to 50% and consume an additional 200 to 300 g of carbohydrate each day in their diet. This eating strategy has been shown to supersaturate carbohydrate stores prior to competition and improve endurance exercise capacity [2, 40]. Thus, the type of meal, amount of carbohydrate consumed, and timing of eating are important factors to maximize glycogen storage and in maintaining carbohydrate availability during training while also potentially decreasing the incidence of overtraining. The ISSN has adopted a position stand on nutrient timing in 2008 [133] that has been subsequently revised [13] and can be summarized with the following points:

1. Intramuscular and hepatic glycogen stores are best maximized by consumption of a high-carbohydrate diet (8–12 g/kg/day). Strategies such as aggressive carbohydrate feedings (~ 1.2 g/kg/hour) that favor high-glycemic (> 70) carbohydrates, addition of caffeine (3–8 mg/kg) and combining a moderate carbohydrate dose (0.8 g/kg/h) with protein (0.2–0.4 g/kg/h) have been shown to promote rapid restoration of glycogen stores.
2. High intensity (> 70% VO_2_Max) exercise bouts that extend beyond 90 min challenge fuel supply and fluid regulation. In these situations, it is advisable to consume carbohydrate at a rate of 30–60 g of carbohydrate/hour in a 6–8% carbohydrate-electrolyte solution (6–12 fluid ounces) every 10–15 min throughout the entire exercise bout. The importance of this strategy is increased when poor feeding or recovery strategies were employed prior to exercise commencement. Consequently, when carbohydrate delivery is inadequate, adding protein may help increase performance, mitigate muscle damage, promote euglycemia, and facilitate glycogen re-synthesis.
3. Consuming a diet that delivers adequate energy (minimum of 27–30 kcal/kg) and protein (1.6–1.8 g/kg/day), preferably with evenly spaced (every 3–4 h) protein feedings (0.25–0.40 g/kg/dose) during the day, should be considered for all exercising individuals.
4. Ingesting efficacious doses (10–12 g) of essential amino acids (EAAs) either in free form or as a protein bolus in 20–40 g doses (0.25–0.40 g/kg/dose) will maximally stimulate muscle protein synthesis (MPS).
5. Pre- and/or post-exercise nutritional interventions (carbohydrate + protein or protein alone) can be an effective strategy to support improvements in strength and body composition. However, the size (0.25–0.40 g/kg/dose) and timing (0–4 h) of a pre-exercise meal may impact the benefit derived from the post-exercise protein feeding.
6. Post-exercise ingestion (immediately-post to 2 h post) of high-quality protein sources stimulates robust increases in MPS. Similar increases in MPS have been found when high-quality proteins are ingested immediately before exercise.

#### Vitamins

Vitamins are essential organic compounds that serve to regulate metabolic and neurological processes, energy synthesis, and prevent destruction of cells. Fat-soluble vitamins include vitamins A, D, E, & K and the body stores fat-soluble vitamins in various tissues, which can result in toxicity if consumed in excessive amounts. Water-soluble vitamins consist of the entire complex of B-vitamins and vitamin C. Since these vitamins are water-soluble, excessive intake of these vitamins are eliminated in urine, with few exceptions (e.g. vitamin B6, which can cause peripheral nerve damage when consumed in excessive amounts). Table 1 describes the RDA, proposed ergogenic benefit, and summary of research findings for fat and water-soluble vitamins. Research has demonstrated that specific vitamins possess various health benefits (e.g., Vitamin E, niacin, folic acid, vitamin C, etc.), while few published studies have reported to find an ergogenic value of vitamins for athletes [134–138]. Alternatively, if an athlete is deficient in a vitamin, supplementation or diet modifications to improve vitamin status can consistently improve health and performance [139]. For example, Paschalis and colleagues [140] supplemented individuals who were low in vitamin C for 30 days and reported these individuals had significantly lower VO_2_Max levels than a group of males who were high in vitamin C. Further, after 30 days of supplementation, VO_2_Max significantly improved in the low vitamin C cohort as did baseline levels of oxidative stress of oxidative stress. Importantly, one must consider that some vitamins may help athletes tolerate training to a greater degree by reducing oxidative damage (Vitamin E, C) and/or help to maintain a healthy immune system during heavy training (Vitamin C). Alternatively, conflicting evidence has accumulated that ingesting high doses of Vitamins C and E may negatively impact intracellular adaptations seen in response to exercise training [141–144], which may consequently negatively impact an athlete’s performance. Furthermore, while optimal levels of vitamin D have been linked to improved muscle health [145] and strength [146] in general populations, research studies conducted in athletes generally fail to report on the ergogenic impact of vitamin D in athletes [147, 148]. However, equivocal evidence from Wyon et al. [149] suggests vitamin D supplementation in elite ballet dancers improved strength and reduced risk for injuries. The remaining vitamins reviewed appear to have little ergogenic value for athletes who consume a normal, nutrient dense diet. Since dietary analyses of athletes commonly indicate that athletes fail to consume enough calories and subsequently may not be consuming adequate amounts of each vitamin, many sport dietitians and nutritionists recommend that athletes consume a low-dose daily multivitamin and/or a vitamin enriched post-workout carbohydrate/protein supplement during periods of heavy training [150]. Finally, athletes may desire to consume a vitamin or mineral for various health (non-performance) related reasons including niacin to elevate high density lipoprotein (HDL) cholesterol levels and decrease risk of heart disease (niacin), vitamin E for its antioxidant potential, vitamin D for its ability to preserve musculoskeletal function, or vitamin C to promote and maintain a healthy immune system.

**Table 1:** Proposed nutritional ergogenic aids – vitamins

Nutrient	RDA	Proposed ergogenic value	Summary of research findings
Vitamin A	Males 900 mcg/d Females 700 mcg/d	Constituent of rhodopsin (visual pigment) and is involved in night vision. Some suggest that vitamin A supplementation may improve sport vision.	No studies have shown that vitamin A supplementation improves exercise performance [139].
Vitamin D	5 mcg/d (age < 51)	Promotes bone growth and mineralization. Enhances calcium absorption. Supplementation with calcium may help prevent bone loss in osteoperotic populations.	Co-supplementation with calcium may help prevent bone loss in athletes susceptible to osteoporosis [707]. However, vitamin D supplementation does not enhance exercise performance [139].
Vitamin E	15 mg/d	As an antioxidant, it has been shown to help prevent the formation of free radicals during intense exercise and prevent the destruction of red blood cells, improving or maintaining oxygen delivery to the muscles during exercise. Some evidence suggests that it may reduce risk to heart disease or decrease incidence of recurring heart attack.	Numerous studies show that vitamin E supplementation can decrease exercise-induced oxidative stress [708–710]. However, most studies show no effects on performance at sea level. At high altitudes, vitamin E may improve exercise performance [711]. Additional research is necessary to determine whether long-term supplementation may help athletes better tolerate training.
Vitamin K	Males 120 mcg/d Females 90 mcg/d	Important in blood clotting. There is also some evidence that it may affect bone metabolism in postmenopausal women.	Vitamin K supplementation (10 mg/d) in elite female athletes has been reported to increase calcium-binding capacity of osteocalcin and promoted a 15–20% increase in bone formation markers and a 20–25% decrease in bone resorption markers suggesting an improved balance between bone formation and resorption [712].
Thiamin (B_1_)	Males 1.2 mg/d Females 1.1 mg/d	Coenzyme (thiamin pyrophosphate) in the removal of CO_2_ from decarboxylic reactions from pyruvate to acetyl CoA and in TCA cycle. Supplementation is theorized to improve anaerobic threshold and CO_2_transport. Deficiencies may decrease efficiency of energy systems.	Dietary availability of thiamin does not appear to affect exercise capacity when athletes have a normal intake [713].
Riboflavin (B_2_)	Males 1.3 mg/d Females 1.7 mg/d	Constituent of flavin nucleotide coenzymes involved in energy metabolism. Theorized to enhance energy availability during oxidative metabolism.	Dietary availability of riboflavin does not appear to affect exercise capacity when athletes have a normal intake [713].
Niacin (B_3_)	Males 16 mg/d Females 14 mg/d	Constituent of coenzymes involved in energy metabolism. Theorized to blunt increases in fatty acids during exercise, reduce cholesterol, enhance thermoregulation, and improve energy availability during oxidative metabolism.	Studies indicate that niacin supplementation (100–500 mg/d) can help decrease blood lipid levels and increase homocysteine levels in hypercholesteremic patients [714, 715]. However, niacin supplementation (280 mg) during exercise has been reported to decrease exercise capacity by blunting the mobilization of fatty acids [716].
Pyridoxine (B_6_)	1.3 mg/d (age < 51)	Has been marketed as a supplement that will improve muscle mass, strength, and aerobic power in the lactic acid and oxygen systems. It also may have a calming effect that has been linked to an improved mental strength.	In well-nourished athletes, pyridoxine failed to improve aerobic capacity, or lactic acid accumulation [713]. However, when combined with vitamins B_1_and B_12,_ it may increase serotonin levels and improve fine motor skills that may be necessary in sports like pistol shooting and archery [717, 718].
Cyano-cobalamin (B_12_)	2.4 mcg/d	A coenzyme involved in the production of DNA and serotonin. DNA is important in protein and red blood cell synthesis. Theoretically, it would increase muscle mass, the oxygen-carrying capacity of blood, and decrease anxiety.	In well-nourished athletes, no ergogenic effect has been reported. However, when combined with vitamins B_1_ and B_6_, cyanocobalamin has been shown to improve performance in pistol shooting [718]. This may be due to increased levels of serotonin, a neurotransmitter in the brain, which may reduce anxiety.
Folic acid (folate)	400 mcg/d	Functions as a coenzyme in the formation of DNA and red blood cells. An increase in red blood cells could improve oxygen delivery to the muscles during exercise. Believed to be important to help prevent birth defects and may help decrease homocysteine levels.	Studies suggest that increasing dietary availability of folic acid during pregnancy can lower the incidence of birth defects [719]. Additionally, it may decrease homocysteine levels (a risk factor for heart disease) [720]. In well-nourished and folate deficient-athletes, folic acid did not improve exercise performance [721].
Pantothenic acid	5 mg/d	Acts as a coenzyme for acetyl coenzyme A (acetyl CoA). This may benefit aerobic or oxygen energy systems.	Research has reported no improvements in aerobic performance with acetyl CoA supplementation. However, one study reported a decrease in lactic acid accumulation, without an improvement in performance [722].
Beta carotene	None	Serves as an antioxidant. Theorized to help minimize exercise-induced lipid peroxidation and muscle damage.	Research indicates that beta carotene supplementation with or without other antioxidants can help decrease exercise-induced peroxidation. Over time, this may help athletes tolerate training. However, it is unclear whether antioxidant supplementation affects exercise performance [709].
Vitamin C	Males 90 mg/d Females 75 mg/d	Used in a number of different metabolic processes in the body. It is involved in the synthesis of epinephrine, iron absorption, and is an antioxidant. Theoretically, it could benefit exercise performance by improving metabolism during exercise. There is also evidence that vitamin C may enhance immunity.	In well-nourished athletes, vitamin C supplementation does not appear to improve physical performance [138, 723]. However, there is some evidence that vitamin C supplementation (e.g., 500 mg/d) following intense exercise may decrease the incidence of upper respiratory tract infections [696, 724, 725].

Recommended Dietary Allowances (RDA) based on the 1989 Food & Nutrition Board, National Academy of Sciences-National Research Council recommendations. Updated in 2001

#### Minerals

Minerals are essential inorganic elements necessary for a host of metabolic processes. Minerals serve as structure for tissue, important components of enzymes and hormones, and regulators of metabolic and neural control. In athletic populations, some minerals have been found to be deficient while other minerals are reduced secondary to training and/or prolonged exercise. Notably, acute changes in sodium, potassium and magnesium throughout a continued bout of moderate to high intensity exercise are considerable. In these situations, athletes must work to ingest foods and fluids to replace these losses, while physiological adaptations to sweat composition and fluid retention will also occur to promote a necessary balance. Like vitamins, when mineral status is inadequate, exercise capacity may be reduced and when minerals are supplemented in deficient athletes, exercise capacity has been shown to improve [151]. However, scientific reports consistently fail to document a performance improvement due to mineral supplementation when vitamin and mineral status is adequate [134, 152, 153]. Table 2 describes minerals that have been purported to affect exercise capacity in athletes. Of the minerals reviewed, several appear to possess health and/or ergogenic value for athletes under certain conditions. For example, calcium supplementation in athletes susceptible to premature osteoporosis may help maintain bone mass [151]. For years, the importance of iron status in female athletes has been discussed [154] and more recent efforts have highlighted that iron supplementation in athletes prone to iron deficiencies and/or anaemia can improve exercise capacity [155, 156]. Sodium phosphate loading can increase maximal oxygen uptake, anaerobic threshold, and improve endurance exercise capacity by 8 to 10% [157]. Increasing dietary availability of salt (sodium chloride) during the initial days of exercise training in the heat helps to maintain fluid balance and prevent dehydration. The American College of Sports Medicine (ACSM) recommendations for sodium levels (340 mg) represent the amount of sodium in less than 1/8 teaspoon of salt and recommended guidelines for sodium ingestion during exercise (300–600 mg per hour or 1.7–2.9 g of salt during a prolonged exercise bout) [158–161]. Finally, zinc supplementation during training can support changes in immune status in response to exercise training. Consequently, several minerals may enhance exercise capacity and/or training adaptations for athletes under certain conditions. However, there is little evidence that boron, chromium, magnesium, or vanadium affect exercise capacity or training adaptations in healthy individuals eating a normal diet. Sport nutritionists and dietitians should be aware of the specialized situations in which different types of minerals may provide support to bolster an athlete’s health or physical performance.

**Table 2:** Proposed nutritional ergogenic aids – minerals

Nutrient	RDA	Proposed ergogenic value	Summary of research findings
Boron	None	Boron has been marketed to athletes as a dietary supplement that may promote muscle growth during resistance training. The rationale was primarily based on an initial report that boron supplementation (3 mg/d) significantly increased β-estradiol and testosterone levels in postmenopausal women consuming a diet low in boron.	Studies which have investigated the effects of 7 wk. of boron supplementation (2.5 mg/d) during resistance training on testosterone levels, body composition, and strength have reported no ergogenic value [280, 281]. There is no evidence at this time that boron supplementation during resistance-training promotes muscle growth.
Calcium	1000 mg/d (ages 19–50)	Involved in bone and tooth formation, blood clotting, and nerve transmission. Stimulates fat metabolism. Diet should contain sufficient amounts, especially in growing children/adole [285] scents, female athletes, and postmenopausal women. Vitamin D needed to assist absorption.	Calcium supplementation may be beneficial in populations susceptible to osteoporosis [726]. Additionally, calcium supplementation has been shown to promote fat metabolism and help manage body composition [727, 728]. Calcium supplementation provides no ergogenic effect on exercise performance.
Chromium	Males 35 mcg/d Females 25 mcg/d (ages 19–50)	Chromium, commonly sold as chromium picolinate, has been marketed with claims that the supplement will increase lean body mass and decrease body fat levels.	Animal research indicates that chromium supplementation increases lean body mass and reduces body fat. Early research on humans reported similar results [285], however, more recent well-controlled studies reported that chromium supplementation (200 to 800 mcg/d) does not improve lean body mass or reduce body fat [287, 291].
Iron	Males 8 mg/d Females 18 mg/d (age 19–50)	Iron supplements are used to increase aerobic performance in sports that use the oxygen system. Iron is a component of hemoglobin in the red blood cell, which is a carrier of oxygen.	Most research shows that iron supplements do not appear to improve aerobic performance unless the athlete is iron-depleted and/or has anemia [729].
Magnesium	Males 420 Females 320	Activates enzymes involved in protein synthesis. Involved in ATP reactions. Serum levels decrease with exercise. Some suggest that magnesium supplementation may improve energy metabolism/ATP availability.	Most well-controlled research indicates that magnesium supplementation (500 mg/d) does not affect exercise performance in athletes unless there is a deficiency [730, 731].
Phosphorus (phosphate salts)	700 mg/d	Phosphate has been studied for its ability to improve all three energy systems, primarily the oxygen system or aerobic capacity.	Recent well-controlled research studies reported that sodium phosphate supplementation (4 g/d for 3 d) improved the oxygen energy system in endurance tasks [504–506]. There appears to be little ergogenic value of other forms of phosphate (i.e., calcium phosphate, potassium phosphate). More research is needed to determine the mechanism for improvement.
Potassium	2000 mg/d^a^	An electrolyte that helps regulate fluid balance, nerve transmission, and acid-base balance. Some suggest excessive increases or decreases in potassium may predispose athletes to cramping.	Although potassium loss during intense exercise in the heat has been anecdotally associated with muscle cramping, the etiology of cramping is unknown [732, 733]. It is unclear whether potassium supplementation in athletes decreases the incidence of muscle cramping [160]. No ergogenic effects reported.
Selenium	55 mcg/d	Marketed as a supplement to increase aerobic exercise performance. Working closely with vitamin E and glutathione peroxidase (an antioxidant), selenium may destroy destructive free radical production of lipids during aerobic exercise.	Although selenium may reduce lipid peroxidation during aerobic exercise, improvements in aerobic capacity have not been demonstrated [734, 735].
Sodium	500 mg/d^a^		During the first several days of intense training in the heat, a greater amount of sodium is lost in sweat. Additionally, prolonged ultraendurance exercise may decrease sodium levels leading to hyponatremia. Increasing salt availability during heavy training in the heat has been shown to help maintain fluid balance and prevent hyponatremia [160, 736].
Vanadyl sulfate (vanadium)	None	Vanadium may be involved in reactions in the body that produce insulin-like effects on protein and glucose metabolism. Due to the anabolic nature of insulin, this has brought attention to vanadium as a supplement to increase muscle mass, enhance strength and power.	Limited research has shown that type 2 diabetics may improve their glucose control; however, there is no proof that vanadyl sulfate has any effect on muscle mass, strength, or power [412, 413].
Zinc	Males 11 mg/d Females 8 mg/d	Constituent of enzymes involved in digestion. Associated with immunity. Theorized to reduce incidence of upper respiratory tract infections in athletes involved in heavy training.	Studies indicate that zinc supplementation (25 mg/d) during training minimized exercise-induced changes in immune function [125, 698, 737, 738].

Recommended Dietary Allowances (RDA) based on the 2002 Food & Nutrition Board, National Academy of Sciences-National Research Council recommendations

^a^Estimated minimum requirement

#### Water

The most important nutritional ergogenic aid for athletes is water and limiting dehydration during exercise is one of the most effective ways to maintain exercise capacity. Before starting exercise, it is highly recommended that individuals are adequately hydrated [162]. Exercise performance can be significantly impaired when 2% or more of body weight is lost through sweat (i.e., a 1.4 kg body weight loss from a 70-kg athlete). When one considers that average sweat rates are reported to be 0.5–2.0 L/hour during exercise and training [128, 162], performance losses due to water loss can occur after just 60–90 min of exercise. Further, weight loss of more than 4% of body weight during exercise may lead to heat illness, heat exhaustion, heat stroke, and possibly death [128]. For this reason, it is critical that athletes adopt a mind set to prevent dehydration first by promoting optimal levels of pre-exercise hydration. Throughout the day and without any consideration of when exercise is occurring, a key goal is for an athlete to drink enough fluids to maintain their body weight. Next, athletes can promote optimal pre-exercise hydration by ingesting 500 mL of water or sports drinks the night before a competition, another 500 mL upon waking and then another 400–600 mL of cool water or sports drink 20–30 min before the onset of exercise. Once exercise commences, the athlete should strive to consume a sufficient amount of water and/or glucose-electrolyte solutions (i.e., sports drinks) during exercise to maintain hydration status. Consequently, to maintain fluid balance and prevent dehydration, athletes need to plan on ingesting 0.5 to 2 L/hour of fluid to offset weight loss. This requires frequent (every 5–15 min) ingestion of 12–16 fluid ounces of cold water or a sports drink during exercise [128, 163–166]. Athletes should not depend on thirst to prompt them to drink because people do not typically get thirsty until they have lost a significant amount of fluid through sweat. Additionally, athletes should weigh themselves prior to and following exercise training to monitor changes in fluid balance and then can work to replace their lost fluid [128, 163–166]. During and after exercise, athletes should consume three cups of water for every pound lost during exercise to promote adequate rehydration [128]. A primary goal soon after exercise should be to completely replace lost fluid and electrolytes during a training session or competition. Additionally, sodium intake in the form of glucose-electrolyte solutions (vs. only drinking water) and making food choices and modifications (added salt to foods) should be considered during the rehydration process to further promote euhydration [167]. Athletes should train themselves to tolerate drinking greater amounts of water during training and make sure that they consume more fluid in hotter/humid environments. Beyond nutrition, allowing one’s physiology the chance to acclimatize to the exercising environment for 10–14 days can help improve heat tolerance and promote thermoregulation. Finally, inappropriate and excessive weight loss techniques (e.g., cutting weight in saunas, wearing rubber suits, severe dieting, vomiting, using diuretics, etc.) are considered dangerous and should be prohibited. Sport nutritionists, dietitians, and athletic trainers can play an important role in educating athletes and coaches about proper hydration methods and supervising fluid intake during training and competition.

### Dietary supplements and athletes

Educating athletes and coaches about nutrition and how to structure their diet to optimize performance and recovery are key areas of involvement for sport dietitians and nutritionists. Currently, use of dietary supplements by athletes and athletic populations is widespread while their overall need and efficacy of certain ingredients remain up for debate. Dietary supplements can play a meaningful role in helping athletes consume the proper amount of calories, macro- and micronutrients. Dietary supplements are not intended to replace a healthy diet. Numerous dietary ingredients have been investigated for potential benefits in an athletic population, to enhance training, recovery and/or performance. Supplementation with these nutrients in clinically validated amounts and at opportune times can help augment the normal diet to help optimize performance or support adaptations towards a training outcome. Sport dietitians and nutritionists must be aware of the current data regarding nutrition, exercise, and performance and be honest about educating their clients about results of various studies (whether pro or con). Currently, misleading information is available to the public and this position stand is intended to objectively rate many of the available ingredients. Additionally, athletes, coaches and trainers need to also heed the recommendations of scientists when recommendations are made according to the available literature and what will hopefully be free of bias. Throughout the next two sections of this paper, various nutritional supplements often taken by athletes will be categorized into three categories: Strong Evidence to Support Efficacy and Apparently Safe, Limited or Mixed Evidence to Support Efficacy, Little to No Evidence to Support Efficacy and/or Safety. Based on the available literature, the resulting classification and analysis focuses primarily on whether the proposed nutrient has been found to affect exercise and/or training adaptations through an increase in muscle hypertrophy and later for the supplement’s ergogenic potential. We recognize that some ingredients may exhibit little potential to stimulate training adaptations or operate in an ergogenic fashion, but may favorably impact muscle recovery or exhibit health benefits that may be helpful for some populations. These outcomes are not the primary focus of this review and consequently, will not be discussed with the same level of detail.

### Convenience supplements

Convenience supplements are commonly found in the form of meal replacement powders (MRP’s), ready to drink supplements (RTD’s), energy bars, and energy gels. These products are typically fortified with vitamins and minerals and differ on the amount of carbohydrate, protein, and/or fat they contain. Uniqueness of these products come from the additional nutrients they contain that are purported to promote weight gain, alter body composition, enhance recovery, and/or improve performance. Most people view these supplements as a nutrient dense snack and/or use them to help control caloric intake when trying to gain and/or lose weight. MRP’s, RTD’s, and energy bars/gels can provide a convenient way for people to meet specific dietary needs and/or serve as good alternatives to fast food, foods of lower nutritional quality, and during times when travel or a busy schedule preclude the ability to consume fresh or other forms of whole food. Use of these types of products are particularly helpful in providing carbohydrate, protein, and other nutrients prior to and/or following exercise to optimize nutrient intake when an athlete doesn’t have time to sit down for a good meal or wants to minimize food volume. Consequently, meal replacements should be used in place of a meal during unique situations and are not intended to replace all meals. Care should also be taken to make sure they do not contain any banned or prohibited nutrients.

### Muscle building supplements

The following section provides an analysis of the scientific literature regarding nutritional supplements purported to promote skeletal muscle accretion in conjunction with the completion of a well-designed exercise-training program. An overview of each supplement and a general interpretation of how they should be categorized is provided throughout the text. Table 3 summarizes how every supplement discussed in this article is categorized. However, within each category all supplements are ordered alphabetically. The reader is encouraged to consider that gains or losses in body masses may positively or negatively impact an individual’s athletic performance. For example, increases in body mass and lean mass are desired adaptations for many American football or rugby players and may improve performance in these activities. In contrast, decreases in body mass or fat mass may promote increases in performance such as cyclists and gymnasts whereby athletes such as wrestlers, weightlifters and boxers may need to rapidly reduce weight while maintaining muscle mass, strength and power.

**Table 3:** Summary of categorization of dietary supplements based on available literature

Category	Muscle building supplements	Performance enhancement
I. Strong Evidence to Support Efficacy and Apparently Safe	• HMB • Creatine monohydrate • Essential amino acids (EAA) • Protein	• β-alanine • Caffeine • Carbohydrate • Creatine Monohydrate • Sodium Bicarbonate • Sodium Phosphate • Water and Sports Drinks
II. Limited or Mixed Evidence to Support Efficacy	• Adenosine-5′-Triphosphate (ATP) • Branched-chain amino acids (BCAA) • Phosphatidic acid	• L-Alanyl-L-Glutamate • Arachidonic acid • Branched-chain amino acids (BCAA) • Citrulline • Essential amino acids (EAA) • Glycerol • HMB • Nitrates • Post-exercise carbohydrate and protein • Quercetin • Taurine
III. Little to No Evidence to Support Efficacy and/or Safety	• Agmatine sulfate • Alpha-ketoglutarate • Arginine • Boron • Chromium • Conjugated linoleic acids (CLA) • D-Aspartic acid • Ecdysterones • Fenugreek extract • Gamma oryzanol (Ferulic acid) • Glutamine • Growth-hormone releasing peptides and Secretogogues • Isoflavones • Ornithine-alpha-ketoglutarate • Prohomones • Sulfo-polysaccharides • *Tribulus terrestris* • Vanadyl sulfate • Zinc-magnesium aspartate	• Arginine • Carnitine • Glutamine • Inosine • Medium-chain triglycerides (MCT) • Ribose

#### Strong evidence to support efficacy and apparently safe

##### β-hydroxy β-methylbutyrate (HMB)

HMB is a metabolite of the amino acid leucine. It is well-documented that supplementing with 1.5 to 3 g/day of calcium HMB during resistance training can increase muscle mass (+ 0.5–1 kg greater than controls during 3–6 weeks of training) and strength particularly among untrained subjects initiating training [168–173] and the elderly [174]. The currently established minimal effective dose of HMB is 1.5 g per day, with 3 g per day offering additional benefits on lean body mass, while 6 g per day do not provide any additional gains in lean mass beyond what was reported with a 3 g dose [169]. To optimize HMB retention, its recommend to split the daily dose of 3 g into three equal doses of 1 g each (with breakfast, lunch or pre-exercise, bedtime) [174]. From a safety perspective, dosages of 1.5–6 g per day have been well tolerated [15, 169, 170]. The effects of HMB supplementation in trained athletes are less clear with selected studies reporting non-significant gains in muscle mass [175–177]. In this respect, it has been suggested by Wilson and colleagues [15] that program design (periodized resistance training models) and duration of supplementation (minimum of 6 weeks) likely operate as key factors. In 2015, Durkalec-Michalski and investigators [178] supplemented highly trained rowers (*n* = 16) in a randomized, double-blind, crossover fashion with either 3 g per day of calcium-HMB or a placebo. Before and after each supplementation period, body composition and performance parameters were assessed. When HMB was provided, fat mass was significantly reduced while changes in lean mass were not significant between groups. The same research group published data of 58 highly trained males athletes who supplemented with either 3 g of calcium-HMB or placebo for 12 weeks in a randomized, double-blind, crossover fashion [179]. In this report, fat mass was found to be significantly reduced while fat-free mass was significantly increased. Finally, Durkalec-Michalski and investigators [180] supplemented 42 highly-trained combat sport athletes for 12 weeks with either a placebo or 3 g of calcium-HMB in a randomized, double-blind, crossover fashion. When HMB was provided, fat-free mass was shown to increase (*p* = 0.049) while fat mass was significantly reduced in comparison to the changes seen when placebo was provided. In conclusion, a growing body of literature continues to offer support that HMB supplementation at dosages of 1.5–3 g for durations as short as three to 4 weeks in untrained populations and longer durations (12 weeks) in trained populations can lead to improvements in fat mass and fat-free mass while participating in various forms of exercise training.

##### Creatine monohydrate

In our view, the most effective nutritional supplement available to athletes to increase high intensity exercise capacity and muscle mass during training is creatine monohydrate. Numerous studies have indicated that creatine supplementation increases body mass and/or muscle mass during training [181, 182]. Body mass increases are typically one to two kilograms greater than controls during 4–12 weeks of training [182]. The gains in muscle mass appear to be a result of an improved ability to perform high intensity exercise enabling an athlete to train harder and thereby promote greater training adaptations and muscle hypertrophy [183–186]. The only clinically significant side effect occasionally reported from creatine monohydrate supplementation has been the potential for weight gain [181, 182, 187, 188]. Although concerns have been raised about the safety and possible side effects of creatine supplementation [189, 190], multiple shorter [191–193] and long-term safety studies have reported no apparent side effects [188, 194, 195] and/or that creatine monohydrate may lessen the incidence of injury during training [196–199]. Consequently, supplementing the diet with creatine monohydrate and/or creatine containing formulations seems to be a safe and effective method to increase muscle mass. The ISSN position stand on creatine monohydrate [10] summarizes their findings as this:

1. Creatine monohydrate is the most effective ergogenic nutritional supplement currently available to athletes in terms of increasing high-intensity exercise capacity and lean body mass during training.
2. Creatine monohydrate supplementation is not only safe, but has been reported to have a number of therapeutic benefits in healthy and diseased populations ranging from infants to the elderly. There is no compelling scientific evidence that the short- or long-term use of creatine monohydrate (up to 30 g/day for 5 years) has any detrimental effects on otherwise healthy individuals or among clinical populations who may benefit from creatine supplementation.
3. If proper precautions and supervision are provided, creatine monohydrate supplementation in children and adolescent athletes is acceptable and may provide a nutritional alternative with a favorable safety profile to potentially dangerous anabolic androgenic drugs. However, it is recommended that creatine supplementation only be considered for use by younger athletes who: a) are involved in serious/competitive supervised training; b) are consuming a well-balanced and performance enhancing diet; c) are knowledgeable about the appropriate use of creatine; and d) do not exceed recommended dosages.
4. Label advisories on creatine products that caution against usage by those under 18 years old, while perhaps intended to insulate their manufacturers from legal liability, are likely unnecessary given the science supporting creatine’s safety, including in children and adolescents.
5. At present, creatine monohydrate is the most extensively studied and clinically effective form of creatine for use in nutritional supplements in terms of muscle uptake and ability to increase high-intensity exercise capacity.
6. The addition of carbohydrate or carbohydrate and protein to a creatine supplement appears to increase muscular uptake of creatine, although the effect on performance measures may not be greater than using creatine monohydrate alone.
7. The quickest method of increasing muscle creatine stores appears to be to consume ~ 0.3 g/kg/day of creatine monohydrate for 5–7 days followed by 3–5 g/day thereafter to maintain elevated stores. Initially, ingesting smaller amounts of creatine monohydrate (e.g., 3–5 g/day) will increase muscle creatine stores over a three to 4 week period, however, the initial performance effects of this method of supplementation are less supported.
8. Clinical populations have been supplemented with high levels of creatine monohydrate (0.3–0.8 g/kg/day equivalent to 21–56 g/day for a 70-kg individual) for years with no clinically significant or serious adverse events.
9. Further research is warranted to examine the potential medical benefits of creatine monohydrate and precursors like guanidinoacetic acid on sport, health and medicine.

##### Essential amino acids (EAA)

Research examining the impact of the essential amino acids on stimulating muscle protein synthesis is an extremely popular area. Collectively, this data indicates that ingesting 6–12 g of the essential amino acids (EAA) in the absence of feeding [200] and prior to [201, 202] and/or following resistance exercise stimulates protein synthesis [202–208], with this response being largely independent of the protein source or food type [209]. Theoretically, this may enhance increases in fat-free mass, but to date limited evidence exists to demonstrate that supplementation with non-intact sources of EAAs (e.g., free form amino acids) while resistance training positively impacts fat-free mass accretion. Moreover, other research has indicated that changes in muscle protein synthesis may not correlate with phenotypic adaptations to exercise training [210]. An abundance of evidence is available, however, to indicate that ingestion of high-quality protein sources can heighten adaptations to resistance training [211]. While various methods of protein quality assessment exist, most of these approaches center upon the amount of EAAs that are found within the protein source, and in nearly all situations, the highest quality protein sources are those containing the highest amounts of EAAs. To this point, a number of published studies are available that state the EAAs operate as a prerequisite to stimulate peak rates of muscle protein synthesis [212–215]. To better understand the impact of ingesting free-form amino acids versus an intact protein source, Katsanos et al. [216] administered similar doses of the essential amino acids (6.72 g) as part of an intact protein (15 g of whey protein isolate) source or as free amino acids while completing a resistance training program in elderly adults. Protein accrual was greater when the amino acid dose was provided in an intact source. While the age of the participants in this study may have impacted outcomes [217], this study’s results do highlight the need for more research to better understand to what extent training adaptations are due to the EAA content or if additional benefits are present from ingesting an intact protein source.

While the EAAs are comprised of nine separate amino acids, some individual EAAs have received considerable attention for their potential role in impacting protein translation and muscle protein synthesis. In this respect, the branched-chain amino acids have been highlighted for their predominant role in stimulating muscle protein synthesis [218, 219]. To this point, Karlsson and colleagues [220] demonstrated significantly higher increases in p70s6k expression in recovery from a single bout of lower-body resistance exercise in seven male participants after ingesting a BCAA solution containing 100 mg/kg BCAA when compared to ingesting a placebo. Interestingly, Moberg and investigators [221] had trained volunteers complete a standardized bout of resistance training in conjunction with ingestion of placebo, leucine, BCAA or EAA while measuring changes in post-exercise activation of p70s6k. They concluded that EAA ingestion led to a nine-fold greater increase in p70s6k activation and that these results were primarily attributable to the BCAAs. Finally, a 2017 study by Jackman et al. [222] compared the ability of a 5.6 g dose of BCAAs (versus a placebo) to stimulate increases in muscle protein synthesis. Myofibrillar muscle protein synthesis rates were increased significantly (~ 20%, *p* < 0.05) in comparison to a placebo. While significant, this magnitude of change was notably less than the post-exercise MPS responses seen when doses of whey protein that delivered similar amounts of the BCAAs were consumed [88, 223]. These outcomes led the authors to conclude that the full complement of EAAs was advised to maximally stimulate increases in MPS.

Of all the interest captured by the BCAAs, leucine is accepted to be the primary driver of acute changes in protein translation. In this respect, Dreyer et al. [224] and others [225] have reported that providing leucine after completion of resistance exercise can further potentiate increases in mTOR signalling and protein translation. In this respect, Jager et al. [11] have highlighted that an ideal dose of leucine to stimulate increases in protein translation is likely somewhere between 1.7–3.5 g.

##### Protein

A growing body of literature is available that suggests higher amounts of protein are needed by exercising individuals to optimize exercise training adaptations [11, 83, 211, 226]. Collectively, these sources indicate that people undergoing intense training with the primary intention to promote accretion of fat-free mass should consume between 1.4–2.0 g of protein per kilogram of body weight per day [83, 226]. Tang and colleagues [95] conducted a classic study that examined the ability of three different sources of protein (hydrolyzed whey isolate, micellar casein and soy isolate) to stimulate acute changes in muscle protein synthesis both at rest and after a single bout of resistance exercise. These authors concluded that all three protein sources significantly increased muscle protein synthesis rates both at rest and in response to resistance exercise. When this response is extrapolated over the course of several weeks, multiple studies have reported on the ability of different forms of protein to significantly increase fat-free mass while resistance training [70, 227–232]. Cermak et al. [211] performed a meta-analysis that examined the impact of protein supplementation on changes in strength and fat-free mass. Data from 22 separate published studies that included 680 research participants were included in the analysis. These authors concluded that protein supplementation demonstrated a positive effect of fat-free mass and lower-body strength in both younger and older participants. Similarly, Morton and investigators [83] published results from a meta-analysis that also included a meta-regression approach involving data from 49 studies and 1863 participants. They concluded that the ability of protein to positively impact fat-free mass accretion increases up to approximately 1.62 g of protein per kilogram of body weight per day whereby higher amounts beyond that do not appear to promote greater gains in fat-free mass. Although more research is necessary in this area, evidence clearly indicates that protein needs of individuals engaged in intense training are elevated and consequently those athletes who achieve higher intakes of protein while training promote greater changes in fat-free mass. Beyond the impact of protein to foster greater training-induced adaptations such as increases in strength and muscle mass, several studies have examined the ability of different types of protein to stimulate changes in fat-free mass [229, 231, 233–235] while several studies and reviews have critically explored the role protein may play in achieving weight loss in athletes [236, 237] as well as during periods of caloric restriction [238, 239]. Therefore, it is simplistic and misleading to suggest that there is no data supporting contentions that athletes need more protein in their diet and/or there is no potential ergogenic value of incorporating different types of protein into the diet. It is the position stand of ISSN that exercising individuals need approximately 1.4 to 2.0 g of protein per kilogram of bodyweight per day [11].

#### Limited or mixed evidence to support efficacy

##### Adenosine − 5′-triphosphate (ATP)

ATP is the primary intracellular energy source and in addition, has extensive extracellular functions including the increase in skeletal muscle calcium permeability and vasodilation. While intravenous administration of ATP is bioavailable [240], several studies have shown that oral ATP is not systematically bioavailable [241]. However, chronic supplementation with ATP increases the capacity to synthesize ATP within the erythrocytes without increasing resting concentrations in the plasma, thereby minimizing exercise-induced drops in ATP levels [242]. Oral ATP supplementation has demonstrated initial ergogenic properties, after a single dose, improving total weight lifted and total number of repetitions [243]. ATP may increase blood flow to the exercising muscle [244] and may reduce fatigue and increase peak power output during later bouts of repeated bouts exercise [242]. ATP may also support greater recovery and lean mass maintenance under high volume training [245], however, this has only been reported in one previous study. In addition, ATP supplementation in clinical populations has been shown to improve strength, reduce pain after knee surgery, and reduce the length of the hospital stay [246]. However, given the limited number of human studies of ATP on increasing exercise-induced gains in muscle mass, more chronic human training studies are warranted.

##### Branched chain amino acids (BCAA)

BCAA supplementation has been reported to decrease exercise-induced protein degradation and/or muscle enzyme release (an indicator of muscle damage) possibly by promoting an anti-catabolic hormonal profile [118, 247, 248] and more recent studies support their ability to favorably promote responses to damaging eccentric muscle contractions [249, 250]. Leucine, in particular, is recognized as a keystone of sorts that when provided in the correct amounts (3–6 g) activates the mTORC1 complex resulting in favorable initiation of translation [251]. To highlight this impact for leucine, varying doses of whey protein and leucine levels were provided to exercising men at rest and in response to an acute bout of lower-body resistance exercise to examine the muscle protein synthetic response. Interestingly, when a low dose of whey protein (6.25 g) was enriched with leucine to equal the leucine content found in a 25-g dose of whey protein, the ability to stimulate muscle protein synthesis was retained. While the 25-g dose of whey protein did favorably sustain the increases in muscle protein synthesis, the added leucine highlights an important role for leucine in stimulating muscle protein synthesis in response to resistance exercise [223]. For these reasons, it has been speculated that the leucine content of whey protein and other high-quality protein sources have been suggested to be primary reasons for their ability to stimulate favorable adaptations to resistance training [252, 253]. Theoretically, BCAA supplementation during intense training may help minimize protein degradation and thereby lead to greater gains in (or limit losses of) fat-free mass, but only limited evidence exists to support this hypothesis. For example, Schena and colleagues [254] reported that BCAA supplementation (~ 10 g/d) during 21-days of trekking at altitude increased fat free mass (1.5%) while subjects ingesting a placebo had no change in muscle mass. Bigard and associates [255] reported that BCAA supplementation appeared to minimize loss of muscle mass in subjects training at altitude for 6 weeks. Finally, Candeloro and coworkers [256] reported that 30 days of BCAA supplementation (14 g/day) promoted a significant increase in muscle mass (1.3%) and grip strength (+ 8.1%) in untrained subjects. Alternatively, Spillane and colleagues [257] reported that 8 weeks of resistance training while supplementing with either 9 g of BCAAs or placebo did not impact body composition or muscle performance. Most recently, Jackman et al. [222] examined the ability of an acute dose of branched-chain amino acids to stimulate increases in muscle protein synthesis. While acute ingestion of BCAAs did promote a 22% greater increase in muscle protein synthesis when compared to a placebo, the determined rates were 50% lower than what is commonly seen when a dose of whey protein containing similar amounts of BCAAs is ingested. As mixed outcomes cloud the ability to make clear determinations, studies strongly suggest a mechanistic role for BCAAs and in particular leucine, yet translational data fails to consistently support the need for BCAA supplementation. Alternatively, multiple studies do support BCAAs ability to mitigate recovery from damaging exercise while their ability to favorably impact resistance training adaptations needs further research. This will be discussed in a later section.

##### Phosphatidic acid

Phosphatidic acid (PA) is a diacyl-glycerophospholipid that is enriched in eukaryotic cell membranes and it can act as a signalling lipid [258]. Interestingly, PA has been repeatedly shown to activate the mammalian target of rapamycin (mTOR) signalling in muscle; an effect which ultimately leads to increases in muscle protein synthesis. For instance, Fang et al. [259] demonstrated that PA activates mTOR in vitro. Hornberger et al. [260] also reported that mechanical stretching of skeletal muscle in situ promotes an increase in intramuscular PA levels and this effect was associated with the activation of mTOR signalling. To date, two chronic human supplementation studies have been performed whereby PA supplementation (750 mg/day) occurred in subjects engaged in resistance training. Hoffman et al. [261] reported that PA supplementation increased whole-body lean body mass (LBM) by 1.7 kg, whereas the placebo group demonstrated no relative change in LBM (0.1 kg; *p* = 0.065 between groups). Joy et al. [262] performed a similar eight-week study with more participants and supervised training sessions, and reported that PA supplementation significantly increased LBM by 2.4 kg, whereas the placebo group demonstrated marginal increases in LBM (1.2 kg; *p* < 0.05 between groups). A third study confirmed the beneficial effects of PA on exercise-induced gains in lean body mass [263]. The currently established dose of PA is 750 mg per day and another study investigating lower doses, 375 and 250 mg per day, failed to show significant benefits on lean body mass [264]. Hence, preliminary human research suggests that PA supplementation can increase anabolic signalling in skeletal muscle and enhance gains in muscle mass with resistance training. Given that PA supplementation studies are in their infancy relative to other muscle-building supplements (e.g., whey protein, creatine, HMB, etc.), future studies are needed in order to determine the optimal dosage, timing, and duration of supplementation needed for optimal muscle mass gains.

### Little to no evidence to support efficacy and/or safety

#### Agmatine sulfate

Agmatine, the decarboxylation product of the amino acid L-arginine, has shown different biological effects in different in vitro and animal models [265] indicating potential benefits in an athletic population. Agmatine is thought to improve insulin release and glucose uptake, assist in the secretion of luteinizing hormone, influence the nitric oxide signalling pathway, offer protection from oxidative stress, and is potentially involved in neurotransmission [266]. It is mostly found in fermented foods [267], with higher levels found in alcoholic beverages. Currently, nearly all research involving agmatine is commonly from animal research models and no human studies have been conducted to examine its impact on blood flow or impacting resistance training adaptations such as strength and body composition. There does not appear to be any scientific evidence that Agmatine supports increases in lean body mass or muscular performance.

#### α-ketoglutarate (α-KG)

α-ketoglutarate (α-KG) is an intermediate in the Krebs cycle that is involved in aerobic energy metabolism and may function to stimulate nitric oxide production. There is some clinical evidence that α-KG may serve as an anticatabolic nutrient after surgery [268, 269]. However, it is unclear whether α-KG supplementation during training may affect training adaptations. Very little research has been conducted on just alpha-ketoglutarate in humans to examine exercise outcomes. For example, Little and colleagues [270] supplemented with creatine, a combination of creatine, α-KG, taurine, BCAA and medium-chain triglycerides, or a placebo. The combination of nutrients increased the maximal number of bench press repetitions completed and Wingate peak power while no changes were reported in the placebo group. Campbell and investigators [271] supplemented 35 healthy trained men with 2 g of arginine and 2 g of α-KG or placebo in a double-blind manner while resistance training for 8 weeks. Supplementation with arginine + α-KG increased bench press strength and Wingate peak power, but did not impact body composition. Finally, Willoughby and colleagues [272] examined the results of arginine α-KG supplementation in relation to increasing nitric oxide production (vasodilation during resistance exercise), hemodynamics, brachial artery flow, circulating levels of l-arginine, and asymmetric dimethyl arginine in active males. This study found that although plasma L-arginine increased, there was no significant impact of supplementation on nitric oxide production after a bout of resistance exercise. Due to the lack of research on α-KG examining its impact on exercise training adaptations, its use cannot be recommended at this time.

#### Arginine

Arginine is commonly classified as a conditionally essential amino acid and has been linked to nitric oxide production and increases in blood flow that are purported to then stimulate enhanced nutrient and hormone delivery and favorably impact resistance training adaptations [273]. To date, few studies have examined the independent impact of arginine on the ability to enhance fat-free mass increases while resistance training. Tang and colleagues [274] used an acute model to examine the ability of an oral 10-g dose of arginine to stimulate changes in muscle protein synthesis. These authors reported that arginine administration failed to impact muscle protein synthesis or femoral artery blood flow. Growth hormone levels did rise in response to arginine ingestion, which contrasts with the findings of Forbes et al., [275] who reported a blunting of growth hormone production after acute ingestion of arginine in strength trained males. Regardless, the Tang study [274] and others [276, 277] failed to link the increase in growth hormone to changes in rates of muscle protein synthesis. Notably, other studies have also failed to show a change in blood flow after arginine ingestion, one of its key purported benefits [272, 278]. Campbell and colleagues published outcomes from an 8 week resistance training study that supplemented healthy men in a double-blind fashion with either a placebo or 2 g of arginine and 2 g of α-ketoglutarate. No changes in fat mass or fat-free mass were reported in this study. Therefore, due to the limited data of arginine supplementation on stimulating further increases of exercise in muscle mass, its use for is not recommended at this time.

#### Boron

Boron is a trace mineral whose physiological role is not clearly understood. A number of proposed functions have been touted for boron: vitamin D metabolism, macromineral metabolism, immune support, increase testosterone levels and promote anabolism [279]. Due to a lack of scientific evidence surrounding boron, no official Daily Reference Intake (DRI) is established. Several studies have evaluated the effects of boron supplementation during training on strength and body composition alterations. However, these studies (conducted on male bodybuilders) indicate that boron supplementation (2.5 mg/d) had no significant impact on muscle mass or strength [280, 281]. Further, two investigations [282, 283] examined the impact of boron supplementation on bone mineral density in athletic and sedentary populations. In both investigations, boron supplementation did not significantly influence bone mineral density. Therefore, due to the limited findings on boron supplementation, its use is not recommended, and more research is warranted to determine its physiological impact.

#### Chromium

Chromium is a trace mineral that is actively involved in macronutrient metabolism. Clinical studies have suggested that chromium potentiates the effects of insulin, particularly in diabetic populations. Due to its close interaction with insulin, chromium supplementation has been theorized to impact anabolism and exercise training adaptations. Initial research was promising with chromium supplementation being associated with increases in muscle and strength, particularly in women [284–286]. Subsequent well-controlled research studies [287] since that time have consistently failed to report a benefit for chromium supplementation (200–800 μg/d for 4–16 weeks) [288–294]. Most recently, chromium supplementation was investigated for its ability to impact glycogen synthesis after high-intensity exercise and was found to exert no impact over recovery of glycogen [295]. In summary, chromium supplementation appears to exert very little potential for its ability to stimulate or support improvements in fat-free mass. Therefore, due to the limited data of arginine supplementation on stimulating further increases of exercise in muscle mass, its use for is not recommended at this time.

#### Conjugated linoleic acids (CLA)

Animal studies indicate that adding CLA to dietary feed decreases body fat, increases muscle and bone mass, has anti-cancer properties, enhances immunity, and inhibits progression of heart disease [296–298]. Although animal studies are impressive [299–301], human studies, at best, suggest a modest ability, independent of exercise or diet changes, of CLA to stimulate fat loss [302–305]. Moreover, very little research has been conducted on CLA to better understand if any scenario exists where its use may be justified. Initial work by Pinkoski et al. [306] suggested that CLA supplementation may help minimize catabolism while resistance training, but overall improvements in body composition from this study failed to yield positive outcomes. Two studies are available that supplemented exercising younger [307] and older individuals [308] with a combination of CLA and creatine and reported significant improvements in strength and body composition, but these results are thought to be the result of creatine. Currently, it seems there is little evidence that CLA supplementation during training can affect lean tissue accretion and has limited efficacy [309].

#### D-aspartic acid

Also known as aspartate, aspartic acid is a non-essential amino acid. Two isomers exist within aspartic acid: L-Aspartic acid and D-Aspartic acid. D-Aspartic acid is thought to help boost athletic performance and function as a testosterone booster. It is also used to conserve muscle mass. While limited research is available in humans examining D-aspartic, Willoughby and Leutholtz [310] published a study to determine the impact of D-aspartic acid in relation to testosterone levels and performance in resistance-trained males. The results showed D-aspartic acid did not impact testosterone levels nor did it improve any aspect of performance. In agreement, Melville and colleagues [311] had participants supplement with either three or 6 g of D-aspartic acid and concluded that neither dose of D-aspartic acid stimulated any changes in testosterone and other anabolic hormones. Later, Melville et al. [312] supplemented 22 men in a randomized, double-blind fashion with either a placebo or 6 g of D-aspartic acid and concluded that 12 weeks of supplementation exerted no impact on resting levels of free or total testosterone and all changes observed in strength or hypertrophy were similar to what was experienced in the placebo group. Based on the currently available literature, D-aspartic acid is not recommended to improve muscle health.

#### Ecdysterones

Ecdysterones (also known as ectysterone, 20 β-Hydroxyecdysterone, turkesterone, ponasterone, ecdysone, or ecdystene) are naturally derived phytoecdysteroids (i.e., insect hormones). They are typically extracted from the herbs *Leuza rhaptonticum* sp., *Rhaponticum carthamoides*, or *Cyanotis vaga*. They can also be found in high concentrations in the herb Suma (also known as Brazilian Ginseng or Pfaffia). Initial interest was generated for ecdysterones due to reports of research from Russia and Czechoslovakia that indicated a potential physiological benefit in insects and animals [313–316]. A review by Bucci on various herbals and exercise performance also mentioned suma (ecdysterone) [317]. Unfortunately, the initial work was available in obscure journals with sub-standard study designs and presentation of results. In 2006, Wilborn and coworkers [318] completed what remains as the only study in humans to examine the impact of ecdysterones while resistance training. Herein, a 200 mg daily dose of 20-hydroxyecdysone over 8 weeks yielded no impact on changes in fat free mass or anabolic/catabolic hormone status. Ecdysterones are not recommended for supplementation to increase training adaptations or performance.

#### Fenugreek extract

Fenugreek (*trigonella foenum-graecum*) is an Ayurvedic herb historically used to enhance masculinity and libido. Fenugreek extract has been shown to increase testosterone levels by decreasing the activity of the aromatase enzyme metabolizing testosterone into estradiol [319, 320]. Initial research by Poole et al. [321] supplemented resistance trained men in a randomized, double-blind fashion with a placebo or 500 mg of Fenugreek extract. After 8 weeks of supplementing and resistance training, significantly greater improvements in body fat, lower body strength, and upper body strength were observed. Wankhede and colleagues [320] reported a significant increase in repetitions performed to failure using the bench press and a reduction in body fat when 600 mg Fenugreek extract was consumed while following a resistance training program. Initial research using Fenugreek extract suggests it may help improve resistance-training adaptations, but more research in different populations is needed before any further recommendations can be made.

#### Gamma oryzanol (ferulic acid)

Gamma oryzanol is a mixture of a plant sterol and ferulic acid theorized to increase anabolic hormonal responses, strength and muscle mass during training [322, 323]. Although data are limited, one study reported no effect of 0.5 g/d of gamma oryzanol supplementation on strength, muscle mass, or anabolic hormonal profiles during 9 weeks of training [324]. Most recently, Eslami and colleagues [325] supplemented healthy male volunteers with either gamma oryzanol or placebo for 9 weeks while resistance training. In this study, changes in body composition were not realized, but a significant increase in strength was found in the bench press and leg curl exercise. With limited research of mixed outcomes at this point, no conclusive recommendation can be made at this time as more research is needed to fully determine what impact, if any, gamma oryzanol supplementation may have in exercising individuals.

#### Glutamine

Glutamine is the most plentiful non-essential amino acid in the body and plays several important physiological roles [74, 326, 327]. Glutamine has been reported to increase cell volume and stimulate protein [328–330] and glycogen synthesis [331]. Despite its important role in physiological processes, there is no compelling evidence to support the use of glutamine supplementation in terms of increasing lean body mass and a 2008 review by Gleeson concluded that minimal evidence is available to support glutamine’s purported role in exercise and sport training [332]. Initial research by Colker and associates [333] reported that subjects who supplemented their diet with glutamine (5 g) and BCAA (3 g) enriched whey protein (40 g) during resistance training promoted about a two pound greater gain in muscle mass and greater gains in strength than ingesting whey protein alone. In contrast, Kerksick and colleagues [232] reported no additional impact on strength, endurance, body composition and anaerobic power of combining 5 g of glutamine and 3 g of BCAAs to 40 g of whey protein in healthy men and women who resistance trained for 10 weeks. In addition, Antonio et al. [334] reported that high-dose glutamine ingestion (0.3 g/kg) offered no impact of the number of repetitions completed using the leg press or bench press exercises. In a well-designed investigation, Candow and co-workers [335] studied the effects of oral glutamine supplementation combined with resistance training in young adults. Thirty-one participants were randomly allocated to receive either glutamine (0.9 g/kg of lean tissue mass) or a maltodextrin placebo (0.9 g/kg of lean tissue mass) during 6 weeks of total body resistance training. The authors concluded glutamine supplementation during resistance training had no significant effect on muscle performance, body composition or muscle protein degradation in young healthy adults. While there may be other beneficial uses for glutamine supplementation (i.e. gastrointestinal health and peptide uptake in stressed populations [336] and, as mentioned previously, mitigation of soreness and recovery of lost force production [337]), there does not appear to be any scientific evidence that it supports increases in lean body mass or muscular performance.

#### Growth hormone releasing peptides (GHRP) and secretagogues

Growth hormone releasing peptides (GHRP) and other non-peptide compounds (secretagogues) facilitate growth hormone (GH) release [338, 339], and can impact sleep patterns, food intake and cardiovascular functioning [340] along with improvements in lean mass in clinical wasting states [341]. These observations have served as the basis for development of nutritionally-based GH stimulators (e.g., amino acids, pituitary peptides, “pituitary substances”, *Macuna pruriens*, broad bean, alpha-GPC, etc.) and continue to capture interest by sporting populations for their potential to impact growth hormone secretion, recovery and robustness of training [342]. Although there is clinical evidence that pharmaceutical grade GHRP’s and some non-peptide secretagogues can increase GH and IGF-1 levels at rest and in response to exercise, it has not been demonstrated that such increases lead to an increase in skeletal muscle mass [343]. Finally, Chromiak and Antonio [344] reported that oral ingestion of many secretagogues fail to consistently stimulate hormone increases in growth hormone and fail to stimulate greater changes in muscle mass or strength. Currently, there is no convincing scientific evidence that secretagogues support increases in lean body mass or muscular performance.

#### Isoflavones

Isoflavones are naturally occurring non-steroidal phytoestrogens that have a similar chemical structure as ipriflavone (a synthetic flavonoid drug used in the treatment of osteoporosis) [345–347]. For this reason, soy protein (which is an excellent source of isoflavones) and isoflavone extracts have been investigated in the possible treatment of osteoporosis as well as their role in body composition changes and changes in cardiovascular health markers. In this respect, multiple studies have supported the ability of isoflavone supplementation in older women alone [348] and in combination with exercise over the course of 6–12 months to improve various body composition parameters [349–351]. Findings from these studies have some applications to sedentary, postmenopausal women. However, there are currently no peer-reviewed data indicating that isoflavone supplementation affects exercise, body composition, or training adaptations in physically active individuals. For example, Wilborn and colleagues [318] reported that 8 weeks of supplementing with isoflavones with resistance training did not significantly impact strength or body composition.

#### Ornithine-α-ketoglutarate (OKG)

OKG (via enteral feeding) has been shown to significantly shorten wound healing time and improve nitrogen balance in severe burn patients [352, 353]. A 2004 review by Cynober postulated that OKG may operate as a precursor to arginine and nitric oxide, but the overall lack of efficacy for arginine and other precursors limits the potential of OKG. Because of its ability to improve nitrogen balance, OKG may provide some value for athletes engaged in intense training. A study by Chetlin and colleagues [354] reported that OKG supplementation (10 g/day) during 6 weeks of resistance training significantly increased upper body strength. However, no significant differences were observed in lower body strength, training volume, gains in muscle mass, or fasting insulin and growth hormone. Since the previously published version of this review, no additional human research has been published and consequently, no further recommendations can be made regarding OKG’s potential as an ergogenic aid.

#### Prohormones and anabolic steroids

Testosterone and growth hormone are two primary hormones in the body that serve to promote gains in muscle mass (i.e., anabolism) and strength while decreasing muscle breakdown (catabolism) and fat mass [355–362]. Testosterone also promotes male sex characteristics (e.g., hair, deep voice, etc.) [356]. Low level anabolic steroids are often prescribed by physicians to prevent loss of muscle mass for people with various diseases and illnesses [363–374]. It is well known that athletes have experimented with large doses of anabolic steroids in an attempt to enhance training adaptations, increase muscle mass, and/or promote recovery during intense training [356–358, 361, 362, 375]. Research has generally shown that use of anabolic steroids and growth hormone during training can promote gains in strength and muscle mass [355, 360, 362, 368, 371, 376–383]. However, a number of potentially life threatening adverse effects of steroid abuse have been reported including liver and hormonal dysfunction, hyperlipidemia (high cholesterol), increased risk to cardiovascular disease, and behavioral changes (i.e., steroid rage) [378, 384–388]. Some of the adverse effects associated with the use of these agents are irreversible, particularly in women [385]. For these reason, anabolic steroids have been banned by most sport organizations and should be avoided unless prescribed by a physician to treat an illness.

Prohormones (e.g., androstenedione, 4-androstenediol, 19-nor-4-androstenedione, 19-nor-4-androstenediol, 7-keto DHEA, and DHEA, etc.) are naturally derived precursors to testosterone or other anabolic steroids. Their use has been suggested to naturally boost levels of these anabolic hormones. While data is available demonstrating increases in testosterone [389, 390], virtually no evidence exists demonstrating heightened training adaptations in younger men with normal hormone levels. In fact, most studies indicate that they do not affect testosterone and that some may actually increase estrogen levels and reduce HDL-cholesterol [378, 389, 391–396]. On a related note, studies have examined the ability of various ingredients to increase testosterone via inhibition of aromatase and 5-alpha-reductase [397]. Rohle et al. [398] and Willoughby et al. [399] reported that significant increases in free testosterone and dihydrotesterone occurred, but soft tissue composition either was not measured [398] or wasn’t changed as a result of supplementation [399]. Consequently, although there may be some potential applications for older individuals to replace diminishing androgen levels, it appears that prohormones have no training value. Since prohormones are “steroid-like compounds”, most athletic organizations have banned their use. Use of nutritional supplements containing prohormones will result in a positive drug test for anabolic steroids. Use of supplements (knowingly or unknowingly) containing prohormones have been believed to have contributed to a number of recent positive drug tests among athletes. Consequently, care should be taken to make sure that any supplement an athlete considers taking does not contain prohormone precursors particularly if their sport bans and tests for use of such compounds. Companies such as Informed Choice (www.informed-choice.org) and National Sanitation Foundation, NSF (aka, NSF Certified for Sport) www.nsf.org) have developed assurance programs to test and screen various nutrition products. Moreover, several professional sporting organizations have incorporated language into the collective bargaining that requires all products provided by teams or sporting organizations must provide products that have achieved certain 3rd party approvals for safety, banned substances and/or label claims. It is noteworthy to mention that many prohormones are not lawful for sale in the USA since the passage of the Anabolic Steroid Control Act of 2004. The distinctive exception to this is dehydroepiandrosterone (DHEA), which has been the subject of numerous clinical studies in aging populations.

#### Sulfo-polysaccharides (myostatin inhibitors)

Myostatin or growth differentiation factor 8 (GDF-8) is a transforming growth factor known as a negative regulator of skeletal muscle hypertrophy [400]. In humans, inhibiting myostatin gene expression has been theorized as a way to prevent or slow down muscle wasting in various diseases, speed up recovery of injured muscles, and/or promote increases in muscle mass and strength in athletes [401]. Since 2010, no additional research has been published that examined the impact of any nutritional ingredient or strategy to inhibit myostatin expression. In humans, myostatin clearly plays a role in regulating skeletal muscle mass. For example, a study by Ivey and colleagues [401] reported that female athletes with a less common myostatin allele experienced greater gains in muscle mass during training and reduced atrophy during detraining. Interestingly, no such changes were reported for men. Willoughby and colleagues [402] supplemented untrained males with 1200 mg/day of *Cytoseira Canariensis* (a form of sea algae), a purported myostatin inhibitor, and reported no changes for fat-free mass, strength and blood concentrations of myostatin. These results were corroborated by Wilborn et al. [318] who reported no impact of sulfo-polysaccaride supplementation on body composition or performance changes. As it stands, there is currently no published data supporting the use of sulfo-polysaccharides or any other ingredient touted to act as a myostatin inhibitor for their ability to increase strength or muscle mass.

#### *Tribulus terrestris*

*Tribulus terrestris* (also known as puncture weed/vine or caltrops) is a plant extract that has been suggested to stimulate leutinizing hormone which stimulates the natural production of testosterone [403]. Consequently, tribulus is marketed as a supplement that can increase testosterone and promote greater gains in strength and muscle mass during training. In human research models, several studies have indicated that tribulus supplementation alone [404, 405] or in combination with other segragotogues and androgen precusors [406, 407] appears to have no effects on body composition or strength during resistance training.

#### Vanadyl sulfate (vanadium)

Vanadyl sulfate is a trace mineral that has been found to affect insulin-sensitivity (similar to chromium) and may affect protein and glucose metabolism [403, 408]. In this regard, reports have highlighted the potential efficacy and support for vanadium to improve insulin sensitivity [409] and assist with the management of diabetes [410]. In relation to its potential ability to impact protein and glucose metabolism, vanadyl sulfate supplementation has been purported to positively impact strength and muscle mass [74, 411]. However, no studies are available that support the ability of vanadyl sulfate supplementation to impact strength or muscle mass in non-diabetic individuals who are currently resistance training [412, 413].

#### Zinc/magnesium aspartate (ZMA)

The main ingredients in ZMA formulations are zinc monomethionine aspartate, magnesium aspartate, and vitamin B-6. ZMA supplementation is based upon the rationale that zinc and magnesium deficiency may reduce the production of testosterone and insulin like growth factor (IGF-1). Consequently, ZMA supplementation is advocated for its ability to increase testosterone and IGF-1, which is further suggested to promote recovery, anabolism, and strength during training. Two studies with contrasting outcomes have examined the ability of acute ZMA administration to increase anabolic hormone concentrations. Initially, Brilla and Conte [414] reported that a zinc-magnesium formulation increased testosterone and IGF-1 (two anabolic hormones) leading to greater strength gains in football players participating in spring training while Koehler et al. [415] reported that ZMA supplementation increased serum zinc and excretion, but failed to change free and total testosterone levels. Wilborn et al. [416] had resistance trained males ingest a ZMA supplement or placebo in a double-blind fashion and resistance train for 8 weeks and found no change in free or total testosterone, strength or fat-free mass (via DXA). It is noted that previous deficiencies in zinc may negatively impact endogenous production of testosterone secondary to its role in androgen metabolism and steroid receptor interaction [417]. To this point, Brilla and Conte [414] did report depletions of both zinc and magnesium, thus increases in testosterone levels could have been attributed to deificient nutritional status rather than a pharmacologic effect. More research is needed to further evaluate the role of ZMA on body composition and strength during training before definitive conclusions can be drawn.

### Performance enhancement supplements

Several nutritional supplements have been proposed to enhance exercise performance. Throughout this section, emphasis is placed upon results that directly measured some attribute of performance. In situations where a nutrient is purported to stimulate increases in fat-free mass and enhance performance (i.e., creatine), a large, more developed section is available while a shorter, more concise section is available in the other category. Table 3 categorizes the proposed ergogenic nutrients into: Strong Evidence to Support Efficacy and Apparently Safe, Limited or Mixed Evidence to Support Efficacy, Little to No Evidence to Support Efficacy and/or Safety.

#### Strong evidence to support efficacy and apparently safe

##### ß-alanine

ß-alanine, a non-essential amino acid, has ergogenic potential based on its role in carnosine synthesis [12]. Carnosine is a dipeptide comprised of the amino acids, histidine and ß-alanine, that naturally occur in large amounts in skeletal muscles. Carnosine is believed to be one of the primary muscle-buffering substances available in skeletal muscle. Studies have demonstrated that taking four to 6 g of ß-alanine orally, in divided doses, over a 28-day period is effective in increasing carnosine levels [418, 419], while more recent studies have demonstrated increased carnosine and efficacy up to 12 g per day [420]. According to the ISSN position statement, evaluating the existing body of ß-alanine research suggests improvements in exercise performance with more pronounced effects on activities lasting one to 4 min; improvements in neuromuscular fatigue, particularly in older subjects, and lastly; potential benefits in tactical personnel [12]. Other studies have shown that ß-alanine supplementation can increase the number of repetitions one can do [421], increase lean body mass [422], increase knee extension torque [423], and increase training volume [421]. In fact, one study also showed that adding ß-alanine to creatine improves performance over creatine alone [424]. While it appears that ß-alanine supplementation can improve performance, other studies have failed to demonstrate a performance benefit [425, 426].

##### Caffeine

Caffeine is a naturally derived stimulant found in many nutritional supplements typically as guarana, bissey nut, or kola. Caffeine can also be found in coffee, tea, soft drinks, energy drinks, and chocolate. Caffeine has also been shown to be an effective ergogenic aid for aerobic and anaerobic exercise with a documented ability to increase energy expenditure and promote weight loss [14]. Research investigating the effects of caffeine on time trial performance in trained cyclists found that caffeine improved speed, peak power, and mean power [427]. Similar results were observed in a recent study that found cyclists who ingested a caffeine drink prior to a time trial demonstrated improvements in performance [428, 429]. Studies indicate that ingestion of caffeine (e.g., 3–9 mg/kg taken 30–90 min before exercise) can spare carbohydrate use during exercise and thereby improve endurance exercise capacity [430, 431]. In addition to the apparent positive effects on endurance performance, caffeine has also been shown to improve repeated sprint performance benefiting the anaerobic athlete [432–434]. Research examining caffeine’s ability to increase maximal strength and repetitions to fatigue are largely mixed in their outcomes. For example, Trexler, et al. [434] reported that caffeine can improve repeated sprint performance but failed to impact maximal strength and repetitions to fatigue using both upper-body and lower-body exercises. In agreement, Astorino and colleagues [435] revealed no change in upper-body and lower-body strength after resistance trained males ingested 6 mg/kg of caffeine. Similarly, Beck and investigators [436] provided resistance trained males with 201 mg caffeine (2.1–3.0 mg/kg) and reported no impact on lower-body strength, lower-body muscular endurance or upper-body muscular endurance. Maximal upper-body strength, however, was improved. In contrast, other studies have indicated that caffeine may favorably impact muscular performance. For example, Goldstein et al. [437] reported that caffeine ingestion (6 mg/kg) significantly increased bench press strength in a group of women but did not impact repetitions to fatigue. Studies by Duncan and colleagues [438–441] have examined the impact of caffeine on strength and endurance performance as well various parameters of mood state while performing maximal resistance exercise. Briefly, these authors have reported improvements in strength and repetitions to failure using the bench press [438, 439] and other exercises [440, 441]. In addition to potential ergogenic impact, these authors also reported that caffeine significantly improved various indicators of mood state [438, 440], lowered ratings of perceived exertion and decreased perception of muscle pain [439, 441] when acute doses of caffeine (5 mg/kg) were provided before maximal resistance exercise. As illustrated, when evaluating the research on caffeine for its ability to impact strength and muscular performance, the findings are equivocal, and, subsequently, more research is needed to better determine what situations may best predict caffeine’s ability to impact strength performance. For example, trained subjects have demonstrated more ergogenic effects compared to untrained subjects [442, 443]. Also, people who drink caffeinated drinks regularly, however, appear to experience less ergogenic benefits from caffeine [444]. Some concern has been expressed that ingestion of caffeine prior to exercise may contribute to dehydration, although several studies have not supported this concern [430, 445, 446]. Caffeine, from anhydrous and coffee sources are both equally ergogenic [434]. Caffeine doses above 9 mg/kg can result in urinary caffeine levels that surpass the doping threshold for many sport organizations. In summary, consistent scientific evidence is available to indicate that caffeine operates as an ergogenic aid in several sporting situations.

##### Carbohydrate

One of the best ergogenic aids available for athletes and active individuals alike, is carbohydrate. Optimal carbohydrate in the diet on a daily basis, in the hours leading up to exercise, throughout exercise and in the hours after exercise can ensure endogenous glycogen stores are maintained and support many types of exercise performance [41–43, 50]. In this respect, athletes and active individuals should consume a diet high in carbohydrate (e.g., 55–65% of calories or 5–8 g/kg/day) to maintain muscle and liver carbohydrate stores [41, 50, 54]. Research has clearly identified carbohydrate as an ergogenic aid that can prolong exercise [41, 68]. For example, Below and colleagues [447] provided research that ingesting carbohydrate throughout a time to exhaustion protocol after nearly an hour of moderate intensity cycling can significantly extend the time cycling is performed. Moreover, Widrick et al. [129] systematically examined all four possible combinations of high and low pre-exercise intramuscular glycogen levels with and without carbohydrate provision before a standard bout of cycling exercise. When carbohydrate was provided, performance was improved. In addition to traditional endurance exercise models, Williams and Hawley [42] summarized the literature involving carbohydrate delivery and performance of team sports that are typically characterized by variable intensities and intermittent periods of heavy exertion and concluded that carbohydrate intake can increase performance. Pochmuller et al. [68] and Colombani et al. [69] have critically pointed to the duration of the involved exercise bout, the intensity of exercise involved, and the fasting status of the individuals as key factors that may impact exercise performance. Further, Burke and colleagues [23, 50], Hawley et al. [43] and Rodriguez et al. [54] have all emphasized the importance of optimal carbohydrate delivery throughout various types of sport and recovery scenarios to support performance. Beyond ingestion, a growing body of literature has drawn attention to the potential impact of carbohydrate mouth rinsing as an ergogenic strategy. Initial work by Carter and colleagues [448] where they demonstrated an increase in time to exhaustion performance while cycling after rinsing (but not swallowing) the oral cavity with a carbohydrate solution versus a no carbohydrate rinse revealed that receptors in the brain might be linked to the mere presence of carbohydrate in the mouth, which subsequently can work to improve various types of exercise performance. While this concept is still emerging, some [449–452] but not all [453–455] of the studies have supported the ability of carbohydrate mouth rinsing to increase performance. Another carbohydrate manipulation strategy has included utilizing high molecular weight carbohydrates solutions, in contrast to traditional low molecular weight beverages, to theoretically accelerate glucose absorption and energy availability. Importantly, the majority of the literature suggests that utilizing a high molecular weight solution can impart changes in oxidized substrates, or patterns of fuel usage, but appears to have no ergogenic effect on performance in males or females [63, 456–459].

##### Creatine monohydrate

As indicated earlier, creatine supplementation is a well-supported strategy to increase muscle mass and strength during training. However, creatine has also been reported to improve exercise capacity in a variety of settings [182, 460–462]. Specifically, and as discussed by Kreider et al. [10], studies have documented improvements in: a) single and multiple sprints, b) work completed across multiple sets of maximal effort, c) anaerobic threshold, d) glycogen loading, e) work capacity, f) recovery, and g) greater training tolerance. Consequently, team sports, individual activities or sports that consist of high intensity, intermittent exercise such as soccer, tennis, basketball, lacrosse, field hockey and rugby can all benefit from creatine use [182]. Moreover, a 2009 study found that in addition to high intensity interval training creatine improved critical power [460]. Less research is available involving creatine supplementation and endurance exercise, but creatine’s ability to promote glycogen loading [463] and storage of carbohydrate [464–466], key fuels during endurance exercise, may translate into improved endurance exercise performance. Indeed, a 2003 study found that ingesting 20 g of creatine for 5 days improved endurance and anaerobic performance in elite rowers [467]. Since creatine has been reported to enhance interval sprint performance, creatine supplementation during training may improve training adaptations in endurance and anaerobic athletes, anaerobic capacity, and allow athletes to complete greater volumes of training at or above anaerobic threshold [468, 469]. Notably, for athletes who struggle to maintain their body mass throughout their competitive season, creatine use may help athletes in this respect. Importantly and in addition to creatine being an effective ergogenic aid in a wide variety of sports, studies have documented these outcomes (improvements in acute exercise capacity, work completed during multiple sets and training adaptations) in adolescents [470–474], younger adults [231, 424, 462, 475–483], and older individuals [484–490]. Regarding creatine and athletic performance, there appears to be a misunderstanding that creatine may result in muscle cramps and dehydration. However, based on many available studies, there is no clinical evidence that creatine supplementation will increase susceptibility of dehydration, muscle cramps, or heat related illness [196, 491].

##### Sodium bicarbonate (baking soda)

During high intensity exercise, acid (H+) and carbon dioxide (CO_2_) accumulate in the muscle and blood. The bicarbonate system is the primary means the body rids itself of the acidity and CO_2_ via their conversion to bicarbonate prior to subsequent removal in the lungs. Bicarbonate loading (e.g., 0.3 g per kg taken 60–90 min prior to exercise or 5 g taken two times per day for 5 days) as sodium bicarbonate has been shown to be an effective way to buffer acidity during high intensity exercise lasting one to 3 min in duration [431, 492–494]. Matson et al. [495] reported improvements in exercise capacity in events like the 400–800 m run while Lindh and colleagues [496] reported that bicarbonate can improve 200 m freestyle swimming performance in elite male swimmers. Similarly, studies have reported the ability of bicarbonate to improve 3 km cycling time trials [497]. Marriott et al. [498] published findings that sodium bicarbonate significantly improved intermittent running performance by 23% and reduced perceived exertion in male team-sport athletes. Interestingly, Percival and investigators [499] reported that sodium bicarbonate supplementation resulted in significantly higher levels of PGC-1-α, a key protein known to drive mitochondrial adaptations. Finally, a meta-analysis by Peart and investigators [500] involving sodium bicarbonate reported the overall treatment effect to be moderate at improving performance with nearly all measured ergogenic outcomes being influenced by the training status of the participants.

In addition, other studies have examined the potential additive benefit of ingesting sodium bicarbonate with either caffeine or beta-alanine. In this respect, Kilding et al. [497] reported significant independent effects of caffeine and bicarbonate on three-kilometer cycling time trial performance, but no additive benefit. Alternatively, Tobias and associates [501] also reported a significant improvement in upper-body power production in trained martial arts athletes after ingesting either beta-alanine or sodium bicarbonate, but noted a distinct synergistic improvement in upper-body power and performance when beta-alanine and sodium bicarbonate were ingested together. In contrast, Danaher et al. [502] had eight healthy males supplement with either beta-alanine, sodium bicarbonate or their combination for 6 weeks in a crossover fashion before completing a repeated sprint ability test while cycling. While buffering capacity was increased, performance was only improved when beta-alanine was provided. Due to the mixed outcomes and relative lack of available studies, more research is recommended examining the synergistic impact of sodium bicarbonate and other ingredients. It is important to highlight that a common complaint surrounding the ingestion of sodium bicarbonate is gastrointestinal distress, thus athletes should experiment with its use prior to performance to evaluate tolerance.

##### Sodium phosphate

Phosphate is best known as an essential mineral found in many common food sources (e.g., red meat, fish, dairy, cereal, etc.) with key functions in bone, cell membranes, RNA/DNA structure and as backbones of phosphocreatine and various nucleotides. In addition, phosphate has been suggested to operate in an ergogenic fashion due to its potential to improve oxygen transport through modulation of 2,3-diphosphoglycerate (DPG) and other lactic-acid-buffering components. Sodium phosphate (NaPO_4_) supplementation has been reported in multiple studies to improve aerobic capacity by 5–12% [503–505], anaerobic threshold by 5–10% [504–507], mean power output [503, 508] and intermittent running performance [509–511]. Collectively these studies have employed a dosing regimen that required 1 g of NaPO_4_ to be taken four times daily for three to 6 days. Not all studies, however [512–514], have reported ergogenic outcomes while factors that impact phosphate absorption, training status and gender posed as potential reasons why supplementation has not universally impacted performance. Brewer and colleagues [513] reported modest (non-significant) effects of NaPO_4_ supplementation on repeated supplementation regimens in trained cyclists completing a time trial. Furthermore, West and investigators [514] used a mixed gender cohort and concluded no change in VO_2_Max resulted after supplementation. Buck et al. [515] were the first to solely examine the impact of NaPO4 in female athletes when they had 13 trained female cyclists complete a 500-kJ time trial after supplementing with either 25, 50, or 75 mg/kg of NaPO_4_ in a randomized, double-blind manner. No significant impact of supplementation was seen at any dosage leading the authors to conclude that females may not respond in the same manner as men. However, the same authors on two occasions [510, 511] examined the impact of NaPO4 in female team sport athletes completing repeated bouts of sprint running and found that NaPO4 significantly improved best and total sprint times when compared to a placebo. Consequently, the impact of gender on the ergogenic potential of NaPO4 remains unclear with consistent benefits in females when repeated sprints are performed but no such benefits during time-trial work.

##### Water and sports drinks

Adopting strategies to limit the loss of body mass due to sweating is critical to maintain exercise performance (particularly in hot/humid environments). People engaged in intense exercise or work in the heat are commonly recommended to regularly ingest water or sports drinks (e.g., 12–16 fluid ounces every 10–15 min) with the overarching goal being to minimize the loss of body mass commonly seen as a result of exercising in a hot and humid environment [516]. Below and colleagues [447] demonstrated the independent ability of both fluid (no carbohydrate) and carbohydrate ingestion to significantly increase cycling performance. Moreover, when the two treatments were combined a synergistic impact on performance was observed. Studies show that ingestion of sports drinks during exercise in hot/humid environments can help prevent dehydration and improve endurance exercise capacity [517–519]. Of note and like carbohydrate, it appears that exercise factors such as the duration and intensity of the exercise bout operate as strong predictors of cycling time-trial performance [520, 521]. Consequently, frequent ingestion of water and/or sports drinks during exercise is one of the easiest and most effective ergogenic aids due to its ability to support thermoregulation and reduce cardiovascular strain during prolonged bouts of exercise, particularly when completed in hot and humid conditions [162, 516].

#### Limited or mixed evidence to support efficacy

##### L-alanyl-L-glutamine

Operating under the same theoretical framework as glutamine, interest in supplementing with L-alanyl-L-glutamine has increased in recent years. The ingredient has two parts: L-alanine and L-glutamine, both of which are amino acids that are mainstays in the transamination processes involving amino acids. Rogero and colleagues [522] supplemented rats with L-alanyl-L-glutamine for the final 21 days of a six-week exercise training program. Supplementation did not impact time to exhaustion performance, but higher levels of glutamine were found when compared to a control group. Cruzat and Tirapequi [523] also reported increases in plasma and intramuscular glutamine along with an improved antioxidative profile in blood, muscle and liver tissue samples of laboratory rats. These results were extended in 2010 to also report an attenuation of inflammation and plasma creatine kinase levels in laboratory rats after exercise training [523].

Since 2010, five peer-reviewed studies have been published using human subjects. Hoffman and colleagues [524] reported, in a group of ten physically active males, that L-alanyl-L-glutamine increased time to exhaustion on a cycle ergometer when exposed to mild dehydration stress. Two years later, the same research group reported that rehydration with L-alanyl-L-glutamine after 2.3% dehydration in a basketball scrimmage led to an improvement in basketball skill performance and visual reaction time when compared to water [525]. A 2016 study indicated that L-alanyl-L-glutamine maintained reaction time in an upper and lower-body activities after an exhaustive bout of treadmill running [526]. Finally, a 2015 paper determined that L-alanyl-L-glutamine significantly improved treadmill running performance when compared to no hydration [527]. Collectively this research indicates that L-alanyl-L-glutamine at dosages ranging 300–1000 mg per 500 mL of fluid can favorably influence hydration status and performance when compared to no fluid ingestion or water only ingestion.

##### Arachidonic acid

Arachidonic acid (ARA) is a long-chain polyunsaturated fatty acid (20:4, n-6) that resides within the phospholipid bi-layer of cell membranes at concentrations that are dependent upon dietary intake [528]. ARA is not found in high amounts in the typical American diet [529]. However, as little as 1.5 g per day of supplementation over a 50-day period has been shown to increase tissue cell membrane stores of ARA [530]. In skeletal muscle, there is evidence that ARA drives some of the inflammatory response to strength training via enhanced prostaglandin signalling [531]. Specifically, exercise liberates ARA from the muscle cell membrane via phospholipase A_2_ activation. Resultant free intracellular ARA is subsequently converted into certain prostaglandins (i.e., PGE_2_ or PGF_2α_) via cyclooxygenase (COX) enzymes [532], and these prostaglandins can signal associated receptors in an autocrine and paracrine manner to up-regulate signalling associated with increases in muscle protein synthesis. Roberts and colleagues [533] were the first group to examine the impact of ARA supplementation on changes in strength and body composition. Over an eight-week period, resistance-trained, college-aged males were supplemented in a double-blind fashion with either a placebo or ARA at a dosage of 1 g per day in conjunction with 90 g/day of whey protein. A significant increase in anaerobic peak power was found in the ARA group, but no other changes in strength or body composition were found. The second study by DeSouza et al. [534] investigated the effects of ARA supplementation (0.6 g/d vs. placebo) in strength-trained college-aged males for 8 weeks with concomitant resistance training and without protein supplementation. These authors reported that lean body mass (2.9%, *p* < 0.05), upper-body strength (8.7%, *p* < 0.05), and anaerobic peak power (12.7%, *p* < 0.05) significantly increased only in the ARA group. Mitchell and colleagues [535] have also published data in 19 resistance-trained men who supplemented, in a double-blind, placebo-controlled fashion, with 1.5 g per day of ARA for 4 weeks and found that ARA supplementation did not impact acute changes in muscle protein synthesis and other mechanistic links to protein translation. The authors concluded that ARA supplementation did not support a mechanistic link between ARA supplementation and short-term anabolism, but may increase translation capacity. Given the limited human data and inconsistent nature (two positive outcomes, one negative outcome) of the findings regarding the efficacy of ARA, it is too early to recommend ARA at this time. In this respect, more chronic human studies testing different doses of ARA supplementation are needed to fully examine its safety and potential efficacy as a performance enhancing or muscle building aid. From a safety perspective and due to ARA being a known pro-inflammatory fatty acid, use of ARA may be contraindicated in populations that have compromised inflammatory health (i.e., inflammatory bowel syndrome, Chron’s disease, etc.).

##### Branched chain amino acids (BCAA)

Ingestion of BCAA (e.g., 6–10 g per hour) with sports drinks during prolonged exercise has long been suggested to improve psychological perception of fatigue (i.e., central fatigue). Accordingly, Mikulski and investigators [536] used 11 endurance trained men to examine the impact of ingesting 16 g of BCAAs and 12 g of ornithine aspartate over a 90-min cycling exercise bout and found that the amino acid combination significantly improved reaction time, but no ergogenic impact was seen when BCAAs were ingested independently. Although a strong rationale and data exist to support an ergogenic outcome, mixed outcomes currently prevail as other studies have failed to report an ergogenic impact of BCAAs [247, 537]. Consequently, more research is needed to fully determine the ergogenic impact, if any, of BCAAs. An important point to highlight surrounding BCAAs is the growing body of literature supporting their ability to mitigate outcomes surrounding muscle damage. In this respect, multiple studies have investigated and offered support for BCAA’s ability to promote recovery, mitigate soreness and attenuate losses in force production [249, 250, 537, 538].

##### Citrulline

Citrulline (2-Amino-5-(carbamoylamino)pentanoic acid or L-Carnitine) is endogenously produced from ornithine and carbamoyl phosphate in the urea cycle. In the body, citrulline is efficiently recycled into arginine for subsequent nitric oxide production through the citrulline-nitric oxide cycle [539]. Unlike arginine, citrulline catabolism is limited in the intestines [540] as well as its extraction from hepatic tissue [541] resulting in the majority of citrulline passing into systemic circulation before conversion to arginine [542]. Due to this and its non-competitive uptake for cell transport [542], oral citrulline supplementation has been shown to be more effective in increasing arginine [543, 544] and activation of nitric oxide synthase (NOS) [544] as well as various biomarkers of nitric oxide [545]. Multiple studies have employed aerobic exercise models to examine citrulline’s impact on performance. Suzuki et al. [546] showed that 2.4 g/day of L-citrulline for 7 days increased plasma nitric oxide metabolites, plasma arginine and 4-km time trial performance. Using a finger flexor exercise model and P31 nuclear magnetic resonance spectroscopy, Bailey and colleagues [547] reported that 7 days of citrulline (6 g/day) significantly increased plasma arginine and nitrite levels and significantly improved VO_2_ kinetics and exercise performance. However, not all studies reported an ergogenic effect whereby Cunniffe et al. [548] reported no impact of 12 g of citrulline malate on the performance of a single bout of high-intensity cycling. In addition to aerobic exercise research, three studies examined the impact of an 8-g citrulline dose while resistance training on various performance outcomes [549–551]. One study [550] evaluated the effects on the number of repetitions performed for chin-ups, reverse chin-ups, and push-ups to failure in trained males. A second study [551] evaluated the effect of citrulline supplementation on the number of repetitions performed for five sequential sets (60% 1RM) to failure on the leg press, hack squat, and leg extension exercises in trained males. The third study [549] evaluated the effects of citrulline supplementation on the number of repetitions performed during six sets each of bench press and leg press exercises to failure at 80% 1RM in trained females. In all three studies, citrulline malate was shown to significantly increase performance during upper- and lower-body multiple-bout resistance exercise performance. Alternatively, Cultrufello and colleagues [552] reported that a 6 g dose of L-citrulline failed to impact both aerobic and anaerobic indicators of exercise performance. The role of malate in combination with citrulline is largely undetermined. Since malate is an important tricarboxylic acid cycle intermediate, this could possibly account for improvements in muscle function [553]. Therefore, it is presently unclear whether these benefits can be solely attributed to citrulline, as well as what role citrulline may play in aerobic and anaerobic performance.

##### Essential amino acids (EAA)

Research exploring the impact of essential amino acids with various forms of exercise has exploded. To date, it is well accepted that ingestion of at least 2 g of the essential amino acid, leucine, is required to stimulate cellular mechanisms controlling muscle hypertrophy [225, 554] and that ingestion of 6–12 g of a complete essential amino acid mixture are needed to maximize muscle protein synthesis [201–208, 555]. However, their impact on performance remains largely unexplored. While sound theoretical rationale exists and multiple acute study designs provide supportive evidence, it is currently unclear whether following this strategy would lead to greater training adaptations and/or whether EAA supplementation would be better than simply ingesting carbohydrate and a quality protein following exercise. Moreover, very little research is available that has examined the ability of EAAs to impact exercise performance. For these reasons, many authors and review articles have encouraged the prioritization of intact protein sources over ingestion of free form amino acids [11, 13, 54, 222] to promote accretion of fat-free mass, but, as mentioned, the impact of this recommendation on performance changes remains undetermined.

##### Glycerol

Ingesting glycerol with water has been reported to increase fluid retention, and maintain hydration status [556–558]. Theoretically, this should help athletes prevent dehydration and improve thermoregulatory and cardiovascular changes. Although studies indicate that glycerol can significantly enhance body fluid, results are mixed on whether it can improve exercise capacity [166, 559–564]. Regarding endurance performance Coutts and investigators [565] had ten trained endurance athletes complete an Olympic distance triathlon under both placebo and glycerol hyperhydration (1.2 g/kg) + 25 mL/kg fluid solution) 2 h before completion of each triathlon and reported that completion time was significantly improved with glycerol hyperhydration over placebo. These findings were corroborated by Goulet et al. [566] when they had six endurance-trained subjects hyperhydrate with glycerol or water 2 h before a prolonged (2 h) bout of cycling at 65% VO_2_max in hot conditions (26-27 °C) followed two-minute intervals at 80% VO_2_max and concluded that glycerol hyperhydration significantly improved performance. In contrast, Marino et al. [567] reported that a similar glycerol hyperhydration protocol did not improve the total distance covered when moderately trained cyclists completed a variable-intensity cycling protocol. Additionally, Goulet et al. [568] combined a hyperhydration strategy (1.2 g/kg glycerol + 26 mL/kg water) 2 h before commencing a two-hour cycling bout at 66% VO_2_max and 25 °C with consuming (500 mL/hour) a sports drink and reported that glycerol hyperhydration failed to impact cardiovascular or thermoregulatory functions as well as endurance performance. McKenna and investigators [569] were one of the only research groups to examine glycerol’s potential to impact anaerobic power after glycerol hyperhydration. After following a double-blind hyperhydration protocol, male collegiate wrestlers lost 3% of their body mass from fluid and completed an anaerobic test where no impact on performance was found. Variable outcomes surrounding glycerol continue to undermine its potential and the ability to offer a recommendation for its use. Consequently, as pointed out by Goulet et al. [556], it is concluded that more research needs to be completed to work through the nuance surrounding glycerol’s potential efficacy, a key point previously summarized by Nelson et al. [570].

##### β-hydroxy β-methylbutyrate (HMB)

For several years, beta-hydroxy-beta-methyl-butyrate (HMB) has received interest for its ability to enhance training adaptations and performance while also delaying or preventing muscle damage [15, 168, 571]. Initial work by Nissen and colleagues [171] showed significant increases in lean body mass and strength with doses 1.5 and 3 g/day in untrained males, with the 3 g dose showing additional benefits over the lower dose. Gallagher and colleagues [169] indicated that a dose of 38 mg/kg/day (approximately 3 g/day) promoted improvements in fat-free mass, peak isometric force and isokinetic torque production, while no changes in maximal strength were seen. In agreement, Thomson and researchers [572] had 22 resistance trained men supplement, in a double-blind fashion, with either HMB or placebo for 9 weeks and concluded that HMB was responsible for a significant increase in lower-body strength. Not all studies, however, have provided support. For example, Kreider et al. [175] used a dose-response, placebo-controlled approach and concluded that three or 6 g of calcium-HMB did not impact body composition or strength adaptations in individuals experienced with resistance exercise after 4 weeks of supplementation and resistance training. Similarly, Hoffman and colleagues [573] reported that HMB supplementation failed to improve anaerobic power production in collegiate football players, a conclusion which aligns with other previous studies [172, 574]. Differences in training regimens (intensities), randomization, and supervision varied in the initial studies and may have contributed to the mixed results. HMB appears to have the greatest effects on performance when training intensity is maximized.

While many of the previous studies have examined, with mixed results, the ergogenic potential of calcium-HMB supplementation in active, recreationally active individuals, Durkalec-Michalski and colleagues completed three investigations [178–180] that all sought to determine the impact of calcium-HMB supplementation in different athlete types. For instance, HMB supplementation (3 g/day) in elite rowers over a 12-week period significantly improved aerobic (VO_2_max, time to reach ventilatory threshold) performance markers and decreased fat mass when compared to changes seen with placebo [178]. Later, Durkalec-Michalski and Jeszka [179] required 58 highly trained males to supplement with calcium-HMB (3 g/day) for 12 weeks. In this study, fat-free mass increased and fat mass decreased along with multiple markers of aerobic capacity when HMB was provided in comparison to a placebo. Most recently, HMB supplementation over 12 weeks in highly-trained combat sport athletes significantly increased (in comparison to placebo) several indicators of aerobic and anaerobic exercise performance [180]. The recent studies by Durkalec-Michalski and colleagues confirmed earlier works by Vukovich [575] and Lamboley [576] that HMB does have a positive effect on increasing aerobic capacity.

HMB is available as calcium-HMB and as free acid. In comparison to calcium HMB, HMB-free acid shows greater and faster absorption (approx. 30 min vs. 2–3 h) [577]. Much of the initial research used calcium-HMB with largely mixed outcomes while studies using the free acid form are more limited. Studies by Wilson and colleagues using the free acid form have indicated robust changes in strength, vertical jump power and skeletal muscle hypertrophy while heavy resistance training alone [578] and in combination with supplemental ATP [579], but others have critically questioned these outcomes [580]. A recent systematic review by Silva and investigators [581] concluded that the free acid form of HMB may improve muscle and strength and attenuate muscle damage when combined with heavy resistance training but stated that more research is needed before definitive conclusions can be determined.

##### Nitrates

Nitrate supplementation has received much attention due to their effects on vasodilation, blood pressure, improved work efficiency, modulation of force production, and reduced phosphocreatine degradation [582–584] all of which can potentially improve sports performance. Nitrate supplementation is most commonly consumed two to 3 h prior to exercise as beetroot juice or sodium nitrate [585] and is prescribed in both absolute and relative amounts ranging from 300 to 600 mg [585] or 0.1 mmol per kilogram of body mass per day, respectively [583, 586]. These dosing amounts appear to be well tolerated when consumed as both supplemental [587] and supplemental sources [588] without significant alterations in hemodynamics or clinical boundaries of hepatorenal and muscle enzyme status [478, 589]. Supplementing highly trained cyclists with sodium nitrate (10 mg per kilogram of body mass) significantly reduced VO_2_peak without influencing time to exhaustion or maximal power outputs [590]. Additionally, 600 mg of nitrate supplementation (given 2 h prior) non-significantly improved the performance of a 500-m time trial performance in elite-level kayak athletes by 2 s [591]. Of practical significance, it should be noted that first place and last place in the 2008 Beijing Olympics, was separated by 1.47 s in the 500-m men’s canoe/kayak flatwater race. Amateur cyclists at simulated altitude (~ 2500 m) observed improved 16.1 km time trial performance with a concomitant decrease in oxygen consumption after beetroot juice (310 mg nitrate) supplementation [592]. Not all findings, however, have reported performance benefits with nitrate supplementation. Nitrate supplementation (~ 385 mg nitrate) 2.5 h before a 50-mile time trial in well-trained cyclists failed to improve performance [593], which was also reported by MacLeod et al. [594] after examining nitrate supplementation (~ 400 mg nitrate) on 10-km time trial performance in normoxia or simulated altitude (~ 2500 m). In well-trained runners, nitrate supplementation (~ 430 mg nitrate) did not improve performance during an incremental exercise test to exhaustion (simulated altitude 4000 m) or a 10-km time trial (simulated altitude, 2500 m) [595] and Nyakayiru et al. [596] reported no impact of nitrate supplementation on changes in VO_2_ and time trial performance in highly trained cyclists. Other studies have also reported an additive or synergistic effects of high-intensity intermittent exercise, endurance exercise, or resistance training when nitrate supplementation is combined with sodium phosphate [511], caffeine [597], or creatine [478], respectively. It is important to mention that dietary nitrates have a health benefit in some, but not all populations [598]. Daily consumption of beetroot juice (~ 320–640 mg nitrate/d) significantly decreased resting systolic blood pressure in older adults by approximately 6 mmHg [599, 600]. Nitrate supplementation (560 mg – 700 mg nitrate) significantly increased blood flow to working muscle and exercise time in older adults with peripheral artery disease [601] as well as significantly improved endothelial function via increased flow-mediated dilation and blood flow velocity in older adults with risk factors of cardiovascular disease [602]. Collectively, these results indicate that nitrate supplementation may improve aerobic exercise performance and cardiovascular health in some populations.

##### Post-exercise carbohydrate and protein

Ingesting carbohydrate with protein following exercise has been a popular strategy to heighten adaptations seen as part of a resistance training program. The rationale behind this strategy centers upon providing an energy source to stimulate MPS via key signal transduction pathways. Additionally, carbohydrate intake will impact insulin status which could promote MPS, limit protein breakdown or both [603–605]. Furthermore, combining carbohydrate with protein can heighten glycogen resynthesis rates, particularly when carbohydrate intake is not optimal [120] and can improve muscle damage responses after exhaustive exercise [606]. A key point for readers to consider when interpreting findings from this literature is the amount of protein, essential amino acids or leucine being delivered by the protein source [11]. In the last few years many studies have agreed that post workout supplementation is vital to recovery and training adaptations [133, 230, 232, 607, 608]. However, the need for adding carbohydrate to protein to maximize hypertrophic adaptations continues to be questioned. For example, Staples and investigators [605] used an acute study design involving stable isotope methodology to investigate the impact of adding 50 g of carbohydrate to 25 g of whey protein ingestion after a single bout of lower body resistance exercise. The authors concluded that the combination of carbohydrate and protein was no more effective at stimulating muscle protein synthesis or blunting rates of muscle protein breakdown than protein alone. Furthermore, Hulmi and colleagues [609] had participants resistance train for 12 weeks and supplement with equivalent doses of whey protein, carbohydrate or whey protein + carbohydrate while having strength and body composition assessed. Overall, changes in strength were similar in all groups while changes in fat-free mass were greater in the protein group when compared to the carbohydrate group. Fat mass was found to significantly decrease in both groups that contained protein in comparison to carbohydrate, but no differences between the two protein-containing groups were noted. In conclusion, these findings underscore the importance of ingesting adequate protein to stimulate resistance training adaptations. Whether or not the addition of carbohydrate can heighten these changes at the current time seems unlikely. This outcome, however, should not distract the reader from appreciating the fact that optimal carbohydrate delivery will absolutely support glycogen recovery, aid in mitigating soreness and inflammation and fuel other recovery demands.

##### Quercetin

Quercetin is a flavonoid commonly found in fruits, vegetables and flowers, and is known for having some health benefits with therapeutic use. In addition, quercetin has been purported in both animal and human models to improve endurance performance. In this respect, Cureton and colleagues [610] supplemented 30 recreationally active, but not highly trained men in a double-blind fashion to ingest either quercetin (1 g/day) or placebo. No changes in total work performed, substrate utilization, or perception of effort were found after supplementation. Similarly, Bigelman and investigators [611] supplemented ROTC cadets with either 1 g of quercetin or a placebo and concluded that VO_2_max was unchanged as a result. These results correspond with the outcomes of other studies that failed to document ergogenic potential for quercetin [612, 613]. In contrast, Nieman et al. [614] supplemented untrained adult males with 1 g of quercetin in a double-blind fashion for 2 weeks and reported that treadmill performance and markers of mitochondrial biogenesis were improved. Similarly, Patrizio et al. [615] used a resistance exercise model and reported quercetin may improve neuromuscular performance while Davis et al. [616] had 12 study participants supplement with either quercetin and placebo and found that quercetin may improve VO_2_max and endurance capacity. A meta-analysis was completed by Pelletier and researchers [617] to summarize the potential impact of quercetin supplementation on endurance performance. This analysis involved seven published studies representing 288 research participants. Only in untrained participants was quercetin found to significantly increase endurance performance. A 2011 meta-analysis by Kressler et al. [618] drew a similar conclusion whereby they indicated quercetin does have benefit, but the size of this effect is trivial and small. Consequently, more research needs to be completed to better identify what situations may exist that support quercetin’s ability to impact exercise performance.

##### Taurine

Taurine is an amino acid found in high abundance in human skeletal muscle [619, 620] derived from cysteine metabolism that plays a role in a wide variety of physiological functions [621–623]. Studies have indicated that training status (higher in trained vs. untrained muscle, reviewed in [624]) and fiber type (higher in type I vs. type II, reviewed in [619]) impact the amount of taurine found in muscle. It has been reported in some [625, 626] but not all studies [627, 628] that taurine may improve exercise performance and mitigate recovery from damaging and stressful exercise [629]. In recent years, many studies have examined the impact of taurine ingestion on various types of exercise performance. In accordance with previous work, ergogenic outcomes related to taurine administration continue to be mixed. Milioni and investigators [628] failed to show an improvement in performance with a 6 g dose of taurine while completing high-intensity treadmill running. Similarly, Balshaw et al. [625] indicated that taurine failed to positively impact 3-km running performance in trained runners. In contrast, a 2017 study by Warnock et al. [630] reported that a 50 mg/kg dose of taurine outperformed caffeine, placebo and caffeine + taurine on performance changes after repeated Wingate anaerobic capacity tests. Finally, a 2018 meta-analysis by Waldron et al. [631] reported that single daily dosages ranging from one to 6 g for up to 2 weeks can significantly improve endurance exercise performance in a range of study participants. Two studies [632, 633] have been completed that examined taurine’s ability to mitigate decrements associated with muscle damage and resistance exercise performance. Notably, oral ingestion at a dosage of 50 mg/kg for 14 days prior to damage and for 7 days after damage significantly increased strength, and decreased soreness and markers of muscle damage [633]. Finally, studies have also supported the ability of taurine to function in an anti-oxidative role, which may promote an improved cellular environment to tolerate exercise stress [634, 635]. While more research continues to be published involving taurine, the consensus of these outcomes continue to be mixed regarding taurine’s potential to enhance physical performance.

#### Little to no evidence to support efficacy and/or safety

##### Arginine

Arginine is known as a conditionally essential amino acid which has been linked with the ability to increase exercise performance, increase growth hormone production, support immune function, increase training tolerance and promote accretion of fat-free mass [273, 636]. Several studies have sought to examine the ergogenic potential of arginine using both endurance and resistance exercise models with largely mixed results. For example, Greer and investigators [637] examined the ability of arginine + alpha-ketoglutarate and reported that the combination did not significantly impact muscle endurance and significantly reduced the number of chin-ups completed. Similar outcomes were found by Aguiar et al. [638] in older women whereby arginine supplementation failed to impact muscle performance. Sunderland et al. [639] supplemented 18 endurance-trained cyclists for 28 days with either arginine (12 g/day) or corn starch and concluded that arginine did not impact VO_2_Max or ventilatory threshold. In accordance, several other studies have failed to positively report on the ability of arginine to operate as an ergogenic aid [278, 640–644]. Alternatively, a few studies have provided evidence of ergogenic potential for arginine. For example, Campbell and researchers [271] supplemented 35 resistance-trained males in a double-blind fashion with arginine (2 g) + alpha-ketoglutarate (2 g) or a placebo and concluded that maximal upper-body strength and wingate peak power were significantly increased after supplementation. Similarly, Bailey and colleagues [645] concluded that acute arginine supplementation reduced the oxygen cost of moderate-intensity exercise and increased tolerance to high-intensity training. Moreover, Pahlavani et al. [646] supplemented male athletes with arginine in a double-blind fashion and concluded that arginine supplementation significantly increased sport performance. As it stands, most of the published literature that has examined the ability of arginine to operate in an ergogenic fashion has failed to report positive outcomes. While more research is certainly indicated, consumers should exercise caution when using arginine to enhance exercise performance.

##### Carnitine

Carnitine is produced endogenously by the liver and kidneys and plays a pivotal role in lipid metabolism. Consequently, many are led to believe that carnitine ingestion will increase the concentration of endogenous carnitine, thereby increasing lipid metabolism and decrease adipose reserves. To date, the majority of the data continues to suggest that carnitine supplementation does not markedly affect muscle carnitine content [647–649], fat metabolism [648, 650, 651], exercise performance [648, 649, 652, 653], or weight loss in overweight [650, 654], obese [651, 655, 656] or trained subjects [657]. For example, Burrus and investigators [658] had ten cyclists ingest combinations of carbohydrate and carnitine while completing a 40-min ride at 65% VO_2_peak before completing an exhaustion ride at 85% VO_2_Peak. No differences in power outputs or times to exhaustion were found with cycling at 85% VO_2_Peak. Of note, studies have suggested that co-ingesting carnitine with carbohydrate can lead to significant increases in intramuscular carnitine [659, 660]. Later, Wall and colleagues [661] reported that endurance exercise performance was improved and improvements in fuel selection appeared to occur. While interesting, more research is needed regarding changes in performance before further recommendations can be made.

##### Glutamine

As outlined above, a strong theoretical framework exists for glutamine’s ability to help an individual tolerate stress, particularly when relying on animal studies. A close examination into the available human research on glutamine makes it more challenging to characterize glutamine’s potential. Theoretically, glutamine supplementation during training should enhance gains in strength and muscle mass, but evidence in this respect has not been consistent. Glutamine supplementation has been shown to improve glycogen stores which could go on to impact certain types of exercise performance [331] and two recent studies suggest that glutamine provision may help support recovery from damaging resistance exercise. In this respect, Street and colleagues [662] concluded that adding glutamine (0.3 g/kg) to a carbohydrate drink significantly improved muscle soreness and force production, but did not impact changes in creatine kinase, when compared to carbohydrate only ingestion. A similar outcome was found by Legault and colleagues [337] who reported that glutamine supplementation significantly lowered perceived soreness levels and led to improved recovery of force production after a damaging bout of eccentric muscle contractions. From an ergogenic perspective, limited research is available, but Antonio et al. [334] reported that 0.3 g/kg glutamine ingestion did not impact the number of repetitions completed with the leg press or bench press exercises. Consequently, minimal research is available to support glutamine’s ability to operate as an ergogenic aid.

##### Inosine

Inosine is a building block for DNA and RNA that is found in muscle. Inosine possesses important roles that may enhance training and/or exercise performance [663]. Although there is some theoretical rationale, available studies indicate that inosine supplementation has no apparent effect on aerobic or anaerobic exercise performance [664–666].

##### Medium chain triglycerides

Medium chain triglycerides (MCT’s) are shorter chain fatty acids known to readily enter the mitochondria and be converted to energy through beta-oxidation [667]. Studies are mixed as to whether MCT’s are ergogenic and can serve as an effective source of fat during exercise [667–671]. A 2001 study found that 60 g/day of MCT oil for 2 weeks did improve running performance [672]. Additionally, Van Zyl and colleagues [671] reported that while MCTs negatively influenced cycling time trial performance when ingested alone in comparison to carbohydrate ingestion, performance was improved when MCTs were combined with carbohydrate. Using a similar exercise model, Goedecke et al. [668] also reported that MCT administration throughout a two-hour moderate intensity cycle ride resulted in a similar performance in completing a 40-km time trial by trained cyclists. A similar outcome was also reported by Vistisen et al. [673]. Beyond equivocal findings, Goedecke et al. [674] and Jeukendrup et al. [667] both reported ergolytic outcomes of MCT administration on sprint performance in trained cyclists and cycling time trial performance, respectively, while the incidence of gastrointestinal complaints increased in both studies. These findings have been confirmed by others that MCT oils are not sufficient to induce positive training adaptations and may cause gastric distress [675, 676]. Consequently, it does not appear likely that MCT favorably impacts acute exercise performance and no evidence exists that training adaptations may be positively impacted either, while multiple studies have reported that MCT ingestion may cause gastrointestinal upset and decrease exercise performance.

##### Ribose

Ribose is a 5-carbon carbohydrate that is involved in the synthesis of adenosine triphosphate (ATP) and other adenine nucleotides. Clinical studies have shown that ribose supplementation can increase exercise capacity in heart patients [677–681] leading to the development of theories that it can operate as ergogenic aid for athletes. Of the available research, most fail to show an ergogenic value for ribose supplementation on exercise capacity in healthy untrained or trained populations [682–684]. A 2006 study [685] investigated the effects of supplementing with either ribose or dextrose over 8 weeks on rowing performance and concluded that ribose was outperformed by the dextrose control [685]. Kreider and associates [684] and Kerksick and colleagues [686] investigated ribose supplementation on measures of anaerobic capacity in trained cyclists and concluded ribose had no positive impact on performance. In 2017, Seifert and investigators [687] had 26 healthy subjects supplement with either 10 g of ribose or 10 g of dextrose for 5 days while completing a single bout of interval exercise and a two-minute power output test. When splitting the participants into high vs. low oxygen uptake levels, the people with low peak VO_2_ experienced significant increases in mean and peak power output along with reductions in ratings of perceived exertion and creatine kinase. No such changes were reported in individuals with high peak VO_2_. As it stands, clinical findings provide support while studies in healthy, trained populations generally fail to report a positive outcome for ribose supplementation. Healthy individuals with lower fitness levels may afford some benefit.

### Supplements to promote general health

In addition to the supplements previously described, several nutrients have been suggested to help athletes stay healthy during intense training. For example, the American Medical Association has recommended that all Americans ingest a daily low-dose multivitamin in order to ensure that people get a sufficient level of vitamins and minerals in their diet [688, 689]. Although daily vitamin and mineral supplementation has not been found to improve exercise capacity in athletes, it may make sense to take a daily vitamin supplement for health reasons. Vitamin D is often recommended to athletes, especially those participating in indoor sports or in cloudy geographies [690]. Although direct evidence linking vitamin D with performance is equivocal, it is clear that vitamin D has a role in regulating immune function, cardiovascular health, and growth and repair. Dosing should be dependent upon baseline levels, which can be measured by any physician [691]. Glucosamine and chondroitin have been reported to slow cartilage degeneration and reduce the degree of joint pain in active individuals which may help athletes postpone and/or prevent joint problems [692, 693]. Meanwhile, other ingredients including undenatured type II collagen (UC-II) may be helpful as well although more research is needed involving athletic applications [694, 695]. Supplemental vitamin C, glutamine, echinacea, quercetin, and zinc have been reported to enhance immune function [125, 696–699]. However, consuming carbohydrate during prolonged strenuous exercise attenuates rises in stress hormones and appears to limit the degree of exercise-induced immune depression [699]. Similarly, although additional research is necessary, vitamin E, vitamin C, selenium, alpha-lipoic acid and other antioxidants may help restore overwhelmed antioxidant defenses exhibited by athletes [700]. One countering argument against higher doses is the potential for these to interfere with adaptive responses to training [699]. Finally, the omega-3 fatty acids docosahexaenoic acid (DHA) and eicosapantaenoic acid (EPA), in supplemental form, are now endorsed by the American Heart Association for heart health in certain individuals stemming from initial scientific statements made in 2002 [701]. This supportive supplement position stems from: 1) an inability to consume cardio-protective amounts by diet alone; and, 2) the mercury contamination sometimes present in whole-food sources of DHA and EPA found in fatty fish. For general health, dosing recommendations range from 3000 mg-5000 mg daily of deep, cold water fish [702]. Consequently, prudent use of these types of nutrients at various times during training may help athletes stay healthy and/or tolerate training to a greater degree.

High intensity exercise can compromise an athlete’s immune health. Infection risk and exercise workload follow a J-Shape curve with moderate intensity exercise reducing the infection risk, and high intensity exercise actually increasing the risk of infection [703]. Immune suppression in athletes further worsens by the psychological stress, foreign travel, disturbed sleep, environmental extremes, exposure to large crowds or an increase exposure to pathogens due to elevated breathing during exercise or competition. Athletes have several nutritional options to reduce the risk and symptoms of upper respiratory tract infections, including probiotics and baker’s yeast beta-glucan. Beta-glucan is a natural gluco polysaccharide derived from the cell walls of highly purified yeast (*Saccharomyces cerevisiae*) and has been shown to significantly decrease upper-respiratory tract infection symptoms in men and women participating in the Carlsbad marathon [704]. Probiotics, often referred to as “friendly” or “good” bacteria, are live microorganisms which when administered in adequate amounts confer a health benefit on the host. An estimated 70% of our immune system is located in your digestive system indicating the importance of a balanced gut microflora on immune health. Probiotics have been shown to reduce the number, duration and severity of upper-respiratory tract infections and gastrointestinal distress in the general population and in athletes, certain strains of probiotics have been shown to significantly reduce the number of upper-respiratory tract infection episodes a well as their severity [705]. Health benefits of probiotics are strain specific and dose dependent, and some strains have failed to show beneficial effects in athletes [706]. Also, consuming carbohydrate during prolonged strenuous exercise attenuates rises in stress hormones and appears to limit the degree of exercise-induced immune depression [699].

## Conclusion

Several factors operate as cornerstones to enhance athletic performance and optimize training adaptations including the consumption of a balanced, nutrient and energy dense diet, prudent training, and obtaining adequate rest. Use of a limited number of nutritional supplements that research has supported to improve energy availability (e.g., sports drinks, carbohydrate, creatine, caffeine, β-alanine, etc.) and/or promote recovery (carbohydrate, protein, essential amino acids, etc.) can provide additional benefit in certain instances. Dietitians and sport nutritionists should stay up to date on current research regarding the role of nutrition on exercise so they can provide honest and accurate information to their students, clients, and/or athletes about the role of nutrition and dietary supplements on performance and training. Furthermore, these professionals should actively participate in exercise nutrition research, write unbiased scholarly reviews for journals and lay publications, and help disseminate the latest research findings to the public. Through these actions, consumers and other professionals can make informed decisions about appropriate methods of exercise, dieting, and/or whether various nutritional supplements can affect health, performance, and/or training. In all situations, individuals are expected and ethically obligated to disclose any commercial or financial conflicts of interest during such promulgations. Finally, companies selling nutritional supplements or promoting exercise, diet or supplementation protocols should develop scientifically based products, conduct research on their products, and honestly market the results of studies so consumers can make informed decisions.

## Abbreviations

1RM: One-repetition maximum
ACSM: American College of Sports Medicine
AER: Adverse Event Reporting
AMA: American Medical Association
ARA: Arachidonic acid
ATP: Adenosine – 5′ – Triphosphate
BCAA: Branched-chain amino acids
cGMP: Good Manufacturing Practices
CHO: Carbohydrate
CLA: Conjugated linoleic acid
CO2: Carbon dioxide
COX: Cyclooxygenase
DHA: Docosahexaenoic acid
DHEA: Dehydroepiandrosterone
DMAA: Dimethylamylamine
DNA: Deoxyribonucleic acid
DPG: Diphosphoglycerate
DRI: Daily Reference Intake
DSHEA: Dietary Supplement Health Education Act
DXA: Dual-energy x-ray absorptiometry
EAA: Essential amino acids
EPA: Eicosapantaenoic acid
FDA: Food and Drug Administration
FDCA: Food, Drug, and Cosmetic Act
FHA: Functional hypothalamic oligomenorrhea/amenorrhea
FTC: Federal Trade Commission
g: Grams
g/hour: Grams per hour
g/kg: Grams of a certain nutrient per kilogram (usually kilograms of body mass)
g/kg/day: Grams of a certain nutrient per kilogram per day (usually per kilogram of body mass)
GDF-8: Growth differentiation factor-8
GES: Glucose-electrolyte solutions
GH: Growth hormone
GHRP: Growth hormone releasing peptides
GPC: Glycerophosphocholine
GRAS: Generally Recognized as Safe
HDL: High density lipoprotein
HMB: β-hydroxy- β-methylbutyrate
IGF-1: Insulin-line growth factor 1
ISSN: International Society of Sports Nutrition
JISSN: Journal of the International Society of Sports Nutrition
Kcals: Kilocalories
Kcals/day: Kilocalories per day
Kcals/kg/day: Kilocalories per kilogram per day (usually per kilogram of body mass)
kg: Kilogram(s)
L/hour: Liters per hour
LBM: Lean body mass
LEA: Low energy availability
MCT: Medium-chain triglycerides
Mg: Milligrams
mL: Millilitres
MLB: Major League Baseball
mmol: Millimoles
MPS: Muscle protein synthesis
MRP: Meal replacement powders
mTOR: Mammalian target of rapamycin
NaPO_4_: Sodium phosphate
NCAA: National Collegiate Athletic Association
NDI: New Dietary Ingredient
NFL: National Football League
NHL: National Hockey League
NHP: Natural Health Product
NLEA: Nutrition Labeling and Education Act
NOS: Nitric oxide synthase
NSF: National Sanitation Foundation
OKG: Ornithine alpha-ketoglutarate
PA: Phosphatidic acid
PDR: Physician’s Desk Reference
PGC-1-α: Peroxisome proliferator-activated receptor gamma coactivator 1-alpha
PRO: Protein
RDA: Recommended daily allowance
RNA: Ribonucleic acid
ROTC: Reserve Officers’ Training Corps
RTD: Ready to drink
U.S.: United States
UC-II: Undenatured type II collagen
VO_2_Max: Maximal volume of oxygen consumption
ZMA: Zinc magnesium aspartate
α-KG: Alpha-ketoglutarate
μg/d: Micrograms per day
